# Supplementary material for: Interfaces govern the structure of angstrom-scale confined water solutions
Source: Nat Commun. 2025 Aug 7;16:7288. doi: 10.1038/s41467-025-62625-w (PMC12332079; doi:10.1038/s41467-025-62625-w)
Supplement: Supplementary file 1 — Supplementary Information [file 41467_2025_62625_MOESM1_ESM.pdf]

Supplementary Information for:

Interfaces Govern the Structure of Angstrom-Scale Confined Water Solutions

Yongkang Wang,[1,2#] Fujie Tang,[3,4,5#] Xiaoqing Yu,[1] Kuo-Yang Chiang,[1] Chun-Chieh Yu,[1] Tatsuhiko Ohto,[6] Yunfei Chen,[2] Yuki Nagata,[1] Mischa Bonn[1*]*

[1] Max Planck Institute for Polymer Research, Ackermannweg 10, 55128 Mainz, Germany.
[2]School of Mechanical Engineering, Southeast University, 211189 Nanjing, China.
[3]Pen-Tung Sah Institute of Micro-Nano Science and Technology, Xiamen University, 361005 Xiamen, China.
[4]Laboratory of AI for Electrochemistry (AI4EC), IKKEM, 361005 Xiamen, China
[5]Institute of Artificial Intelligence, Xiamen University, 361005 Xiamen, China
[6]Graduate School of Engineering, Nagoya University, Nagoya 464-8603, Japan.
[#]Yongkang Wang and Fujie Tang contributed equally to this work.
[*]Correspondence to: nagata@mpip-mainz.mpg.de, bonn@mpip-mainz.mpg.de

Supplementary Information contains:

Supplementary Methods Sections 1-12

Supplementary Discussion Sections S1 to S15

Figs. S1 to S37

Table S1

Contents

1. Chemicals 3
2. Substrate Preparation 3
3. Nanoconfined Water Sample Preparation 3
4. Suspended Graphene on the Water Surface 4
5. Sample Cell 5
6. Raman Measurement 6
7. HD-SFG Measurement 6
8. Calibration of Experimental $\chi^{(2)}$ Spectra 7
9. Phase measurement 8
10. *Ab Initio* Molecular Dynamics (AIMD) Simulation 12
11. Machine Learning Force Field MD Simulation 15
12. $\chi^{(2)}$ Spectrum Calculation 17

Supplementary Discussion 21

S1. LiCl Concentration and Its Effect on SFG Spectra 21
S2. Characterization of the CaF_2 Surface 26
S3. AFM Characterization of the Sample Height 27
S4. Raman Characterization of the Graphene Sheet 28
S5. Capillary Condensation 30
S6. SFG Spectra at ppp Polarization 32
S7. Amplitude Calibration of the SFG Spectra at ssp Polarization 33
S8. Extraction of $\chi^{(2)}_{yyz}$ from $\chi^{(2)}_{ssp,\text{ eff}}$ 38
S9. Real part of $\chi^{(2)}_{yyz}$ 40
S10. Structural Dynamic Information of Water Computed from AIMD Data 41
S11. Solubility of LiCl Ions under Nanoconfinement 44
S12. Dependence of Water Thickness in Nanoconfined System on SFG Spectra 45
S13. SFG Spectra Computed from AIMD Data 46
S14. SFG Spectra Computed from AIMD and MLFF-MD Trajectories 47
S15. The Ions Distributions of the Nanoconfined System from MLFF-MD Trajectories 48

Supplementary References 49

Supplementary Methods

1. Chemicals

All related chemicals of lithium chloride (LiCl), potassium chloride (KCl), heavy water (D_2O), hydrochloride (HCl, 37%), concentrated sulfuric acid (H_2SO_4, 98%), 30 wt. % hydrogen peroxide solution (H_2O_2), ammonium persulfate ($(NH_4)_2S_2O_8$), cellulose acetate butyrate (CAB), ethyl acetate, 2-propanol, and acetone were purchased from Sigma-Aldrich and were all of the analytical grade without further purification. Deionized water was provided by a Milli-Q system (resistivity ≥ 18.2 MΩ·cm and TOC ≤ 4 ppb) and was saturated with argon by bubbling gas through it for 30 minutes before use. CVD-grown graphene on copper foils was purchased from Grolltex Inc.

2. Substrate Preparation

CaF_2 substrates (25 mm diameter with a thickness of 2 mm, Korth Crystals GmbH) and SiO_2 substrates (25 mm diameter with a thickness of 2 mm, PI-KEM Ltd), and water-free SiO_2 substrates (10×10×1 mm^3, PI-KEM Ltd) were cleaned with acetone and 2-propanol sequentially. Ultrasonic cleaning was avoided to guarantee a flat CaF_2 surface. Note that the facet of the CaF_2 and SiO_2 substrates is unclear. After that, 100 nm-thick gold film was thermally evaporated onto the CaF_2 and SiO_2 substrates with a shadow mask. The gold film serves as the reference sample to generate a stable and precise reference phase for the HD-SFG measurements. Additionally, the gold film also serves as a marker for the AFM and HD-SFG measurements.

3. Nanoconfined Water Sample Preparation

The nanoconfined water samples were prepared by using the wet transfer technique[1,2] to enclose water between a graphene sheet and a CaF_2 substrate (Fig. S1). In brief, we used a commercial CVD-grown large-area monolayer graphene sheet on copper foil (Grolltex Inc). The graphene sheet was exposed to LiCl solution and was then transferred onto the CaF_2 substrate. A thin film of LiCl solution was trapped between the graphene and the substrate due to the hydrophilic nature of the CaF_2 substrate. We also prepared the samples using KCl and all reported results were similar (See Fig. S10). Upon drying for ~12 hours in air with an RH of ~25%, nanoconfined water samples were obtained as a result of capillary condensation[3] (see Supplementary Discussion S5 for more details). Finally, the CAB layer was removed by immersing the sample in acetone. Before assembling the sample onto the sample cell, the prepared sample was allowed to be stored for ~6

hours at RH ~25% for evaporation of acetone. Using this method, a centimeter-sized nanoconfined water sample was obtained. We fabricated the capillary devices with graphene and SiO_2 using the same procedures.

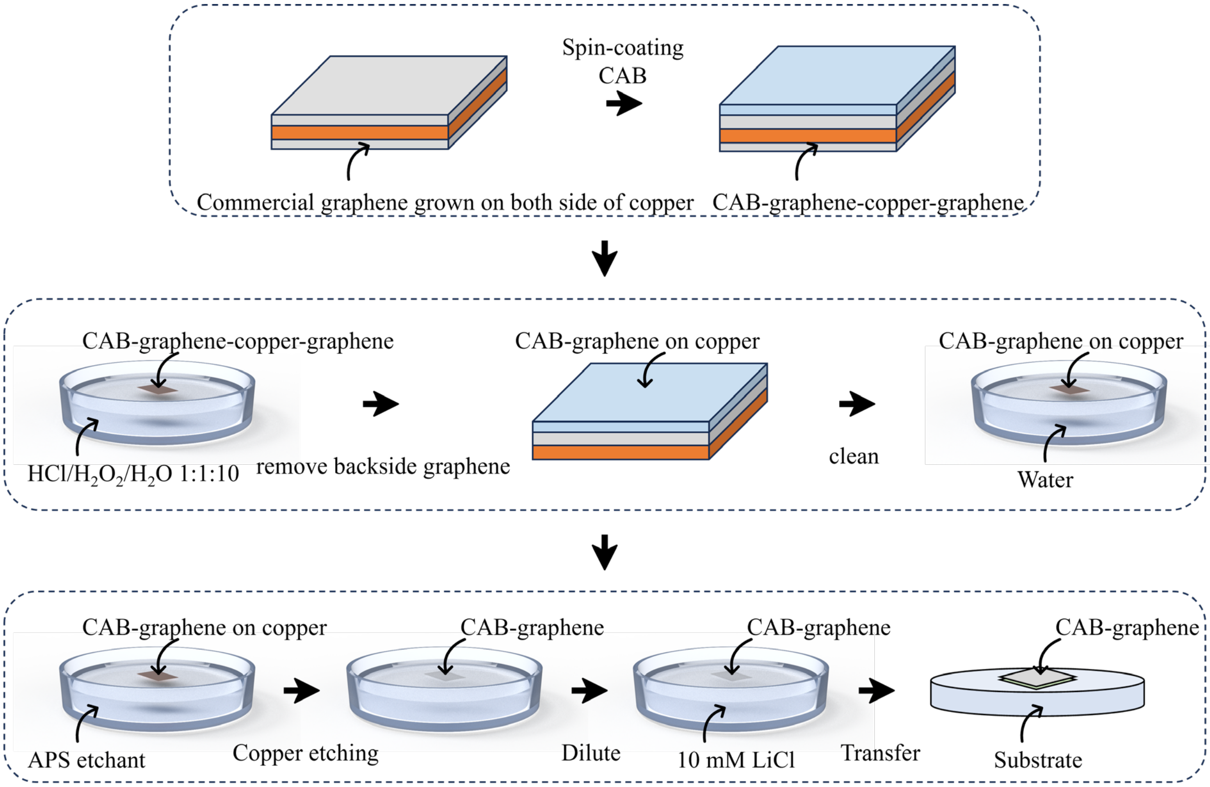

Fig. S1. Procedures for the preparation of nanoconfined water sample.

4. Suspended Graphene on the Water Surface

The preparation of the suspended graphene on the water surface was similar to Refs.[4–6]. Detailed procedures are summarized in Fig. S2. In brief, the graphene layer grown on the backside of the copper foil was first removed using $HCl/H_2O_2/H_2O$ mixture solution. Then, the CAB-coated graphene on the copper foil was immersed in acetone to remove the CAB layer. Subsequently, the copper foil was exposed to a 10 mM APS solution for more than 12 hours to etch away the copper foil. Then the solution was recycled with pure water several times to dilute and remove the etching agents. Finally, the solution was replaced by the electrolyte (LiCl) solution to reach the target concentrations. An O-ring was used to trap the prepared graphene sample. Using this method, centimeter-sized monolayer graphene suspended on the water surface was obtained (Fig. S2).

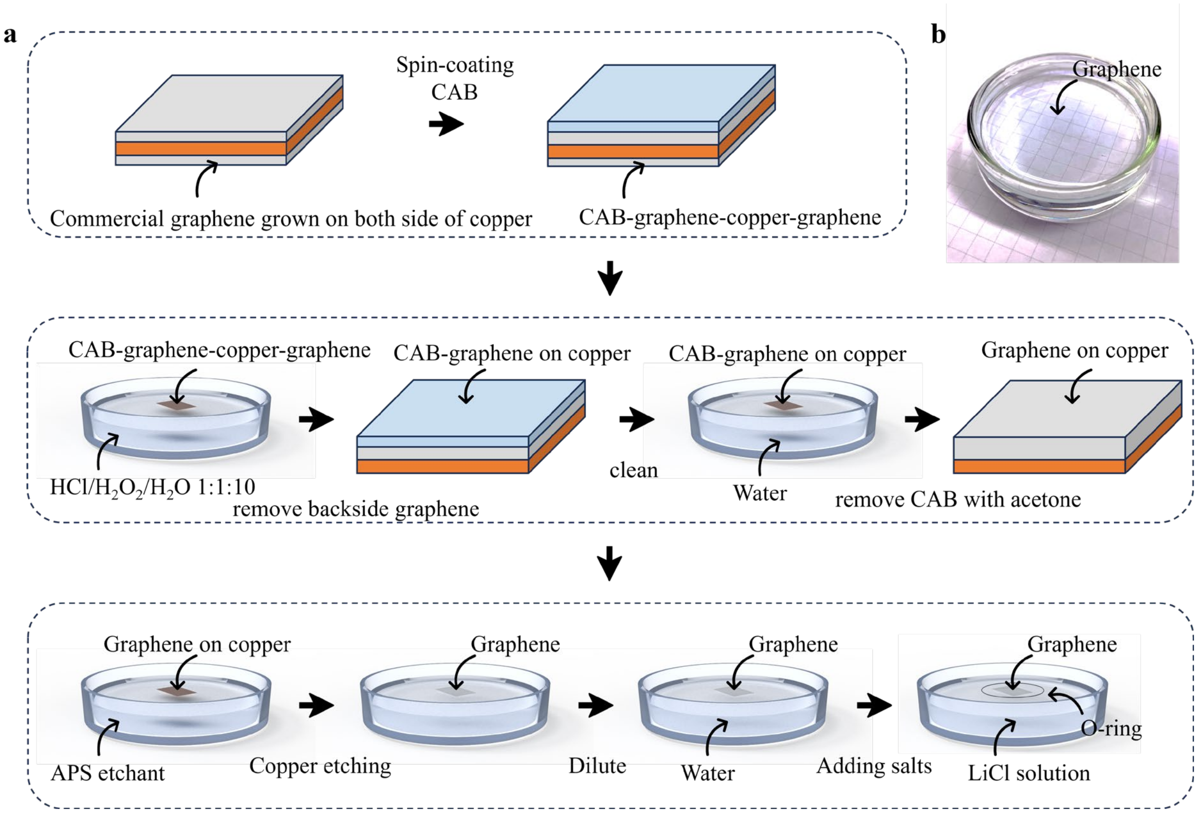

Fig. S2. Preparation of suspended graphene on the water surface. a. Sample preparation procedures. **b.** A photo of the centimeter-sized suspended graphene on the water surface. The diameter of the petri dish is 5 cm.

5. Sample Cell

Our sample cell is schematically depicted in Fig. 1f and Fig. S3a. A photo of the cell is shown in Fig. S3b. The cell mainly consists of two rectangular polytetrafluoroethylene (PTFE) parts, the top clamp part, and the bottom flowing channel (~12×3×3 mm^3) part. The top clamp has an opening of ~16 mm in diameter for the light beam paths. The bottom part has two round holes on the left- and right-side walls, serving as the inlet and outlet of nitrogen (N_2), respectively. Another hole near the cell outlet was connected to a humidity sensor to measure the RH inside the sample cell. The prepared sample and an O-ring were then sandwiched between the top and the bottom PTFE parts. The O-ring creates a seal between the N_2 or aqueous solution and the air. The base and clamp parts were cleaned with piranha solution before use.

The RH in the cell was tuned by purging the cell with N_2 of different RHs. The RH of the N_2 was tuned by changing the relative flow rate of a mixture comprising dry N_2 and wet N_2 while

maintaining the overall N_2 flow rate in the sample cell (Fig. S3c). To guarantee the RH control, a microscopic humidity sensor was connected to the sample cell near the cell outlet to measure the RH in the sample cell (Fig. S3b).

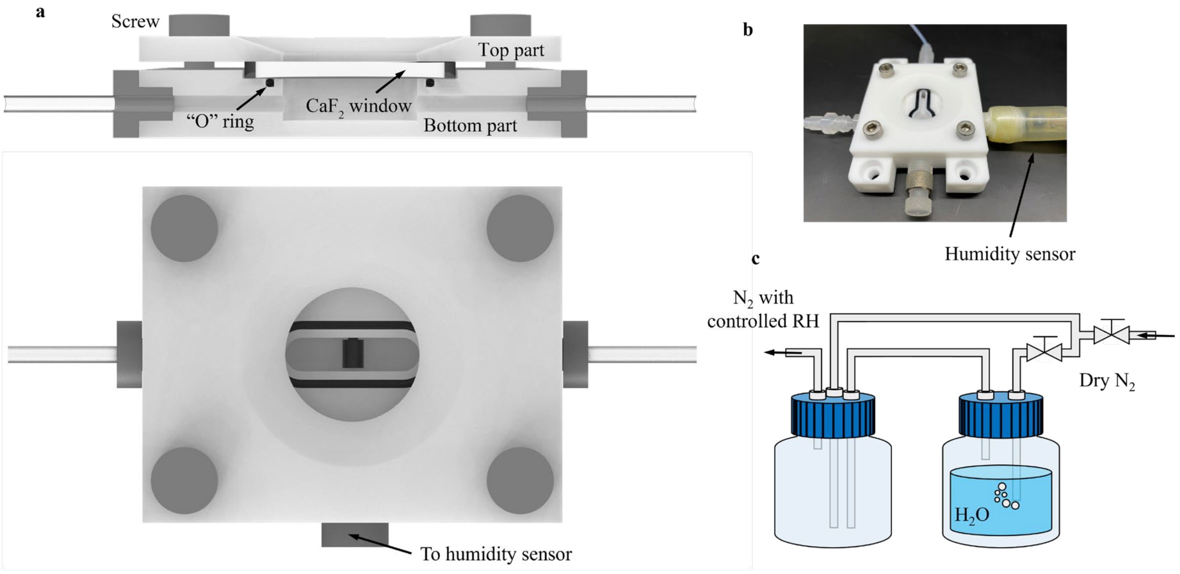

Fig. S3. Experimental setup. a. Schematic diagram of the sample cell for HD-SFG measurements. **b.** A photo of the sample cell. **c.** Tuning RH of N_2.

6. Raman Measurement

The Raman spectra were recorded with a WITec confocal Raman spectrometer (Alpha 300 R, ×10 objective) with 600 grooves/mm grating, 532 nm laser, 2 mW power, and 10 s integration time.

7. HD-SFG Measurement

HD-SFG measurements were performed on an HD-SFG setup in a non-collinear beam geometry with a Ti: Sapphire regenerative amplifier laser system. A detailed description can be found in Refs.[7,8]. HD-SFG spectra were measured in a dried air atmosphere to avoid spectral distortion due to water vapor. The sample cell was purged with N_2 of different RHs during the measurement to tune the thickness of the nanoconfined water. We checked the sample height with a displacement sensor (CL-3000, Keyence with a resolution of ~0.1 μm). The IR, visible, and LO beams were directed at the sample at incidence angles of 33°, 39°, and 37.6°, respectively. The measurements were performed at the *ssp* polarization combination, where *ssp* denotes *s*-polarized SFG, *s*-polarized visible, and *p*-polarized IR beams.

For the nanoconfined water sample and suspended graphene sample, each spectrum was acquired with an exposure time of 2 minutes and measured fifteen times on average with a total exposure time of 30 minutes. The power of the IR and visible beams was reduced to below 3 mW to avoid burning the graphene sheet. For the CaF_2/water interface, the power of the IR and visible beams was around 4 mW and 8 mW, respectively. Each spectrum was acquired with an exposure time of 10 minutes and measured 6 times on average with a total exposure time of 60 minutes. The nanoconfined water sample and CaF_2/water sample HD-SFG spectra at *ssp* polarization were normalized with that for the CaF_2/gold at *ssp* polarization. The suspended graphene sample HD-SFG spectra at *ssp* polarization were normalized with that for the air/*z*-cut quartz (zqz) at *ssp* polarization.

8. Calibration of Experimental $\chi^{(2)}$ Spectra

The total intensity of the HD-SFG signal is represented by the sum of sample sum frequency (SF) light and local oscillator (LO) field reflected at an interface, which can be expressed as follows:

$$|E_{\mathrm{total}}|^2 = \left|E_{\mathrm{sample}}\right|^2 + |E_{\mathrm{LO}}|^2 + E_{\mathrm{sample}}E_{\mathrm{LO}}^{*}e^{i\omega T} + E_{\mathrm{sample}}^{*}E_{\mathrm{LO}}e^{-i\omega T}, \tag{S1}$$

where E_{sample} and E_{LO} are the electric fields of the SF light from the sample and the LO, respectively. T is the time delay between SF lights from the sample SF and the LO fields. * represents the complex conjugate. The third term on the right-hand side of Eq. S1 was picked up using time-domain filtration (A combination of a boxcar function given by Eq. S2 with t_c the cutoff time and a Happ-Genzel function given by Eq. S3)[9] with Fourier transform and was then converted to an interferogram through inverse Fourier transform[10]. The measured complex-valued spectra of second-order nonlinear susceptibility ($\chi^{(2)}_{ssp,\,\mathrm{measured}}$) of the nanoconfined water sample were obtained via the Fourier analysis of the obtained interferogram and normalization with that of the CaF_2/gold interface (Eq. S4).

$$A(t) = \begin{cases} 1 \text{ for } t \geq t_c \\ 0 \text{ for t} < t_c \end{cases}, \tag{S2}$$

$$A(t) = 0.54 + 0.46\cos\left(\frac{\pi(\mathrm{t} + 0.9\ \mathrm{ps})}{0.5\ \mathrm{ps}}\right), \tag{S3}$$

$$\chi^{(2)}_{ssp,\,\mathrm{measured}} = \frac{E_{\mathrm{sample}}E_{\mathrm{LO}}^{*}e^{i\omega T}}{E_{\mathrm{gold}}E_{\mathrm{LO}}^{*}e^{i\omega T}}, \tag{S4}$$

9. Phase measurement

Our phase measurement methods adhere to standard procedures widely used by various groups[11–13]. Briefly, signals from the target sample (nanoconfined water sample and CaF_2/water) and reference (CaF_2/gold) were alternately collected by translating the sample along the *y*-axis (indicated in Fig. 1a). To minimize propagation phase difference caused by variations in CaF_2 thickness, we used a CaF_2 substrate with optically polished surfaces on both sides to ensure its flatness. Furthermore, the gold layer was intentionally coated near the sample position, allowing both sample and reference spectra to be measured by moving the sample only a few hundred micrometers (~300 μm, as shown in Fig. 1a). This limited movement corresponds to a negligible thickness change of ~0.8 nm (calculated based on flatness, e.g., λ/10 at 633 nm), resulting in a propagation phase difference of less than 1°, which is insignificant.

To further demonstrate the reproducibility of both the measured water signal and the phase, in response to the reviewer's comment, we present the $\mathrm{Im}\chi_{ssp}^{(2)}$ spectra and the corresponding phase spectra obtained from six different samples measured at the indicated times in Fig. S4. These results validate the reproducibility of the experimental data as well as the stability and accuracy of the phase measurements.

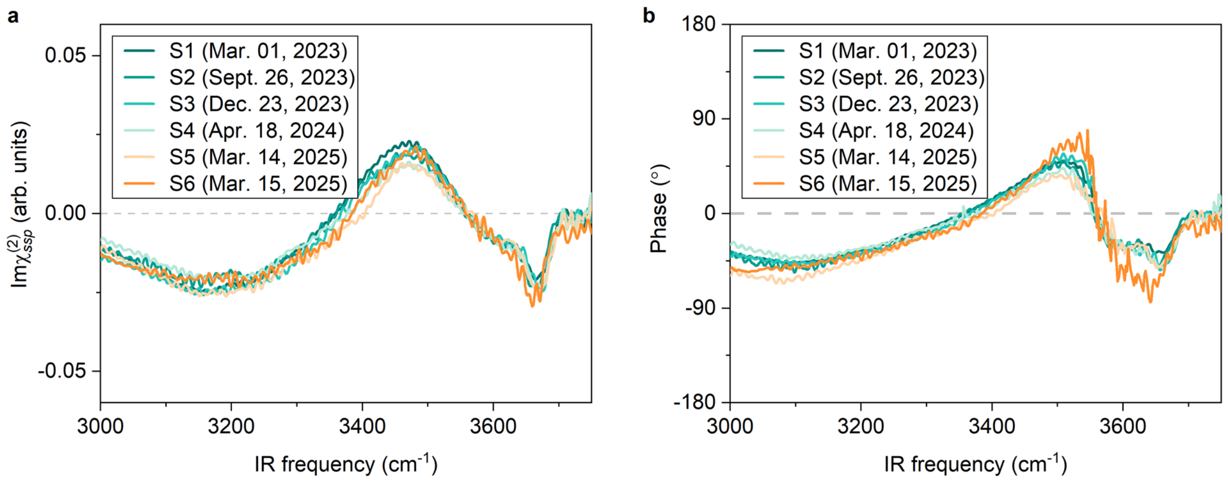

Fig. S4. Experimental reproducibility. **a**. Experimental $\mathrm{Im}\chi_{ssp}^{(2)}$ spectra of the nanoconfined water measured from different samples. **b**. Corresponding phase spectra. The grey dashed lines in (**a**) and (**b**) serve as zero lines.

We determine the phase of the gold film by measuring the O-H stretching Im$\chi^{(2)}$ spectrum of D_2O at the interface via normalization of the signal with that of the CaF_2/gold sample. As D_2O does not have any vibrational response in the O-H stretching region[14], we can determine the phase of the gold surface by the fact that the Im$\chi^{(2)}$ spectrum shows a flat zero line[8].

The non-resonant signal from D_2O (liquid) is weak compared to gold. Therefore, in our HD-SFG measurement, we intentionally set the local oscillator (LO) signal intensity from ZnO in between the D_2O and gold by tuning the position of ZnO (relative to the focus plane) to generate appropriate interference for both the measurement of D_2O and gold. The typical interference spectra in the O-H stretching region for the CaF_2/gold interface and the CaF_2/D_2O interface are shown in S5a. The $\chi^{(2)}_{yyz}$ signals are shown in Fig. S5b. To improve the signal-to-noise ratio, the $\chi^{(2)}_{yyz}$ signal was measured three times for an average. The $\chi^{(2)}_{yyz}$ signals remain spectrally flat across the O-H stretching region. The corresponding phase spectrum (Fig. S5c) exhibits a stable and constant phase with a S.D. of approximately ±2.4°, calculated from the data points across the O-H stretching region. Our experimental results are consistent with previous intensity measurements demonstrating that gold film signals are spectrally flat in this region, similar to those of *z*-cut quartz (commonly used as a standard phase and intensity reference in HD-SFG spectroscopy)[15,16]. These findings indicate that the phase of gold shows minimal frequency dependence and remains nearly constant across the O-H stretching frequency region, consistent with previous phase measurements[17,18].

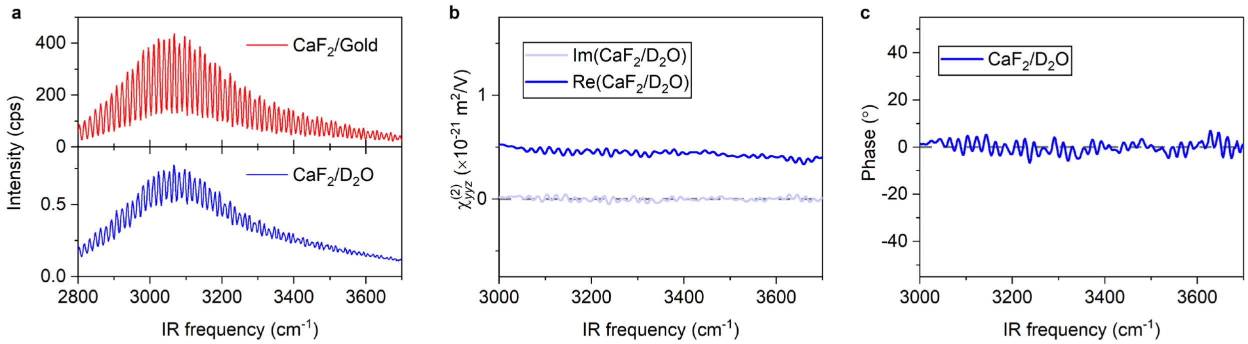

Fig. S5. Phase measurement. a. Interference spectra in the O-H stretching region for the CaF_2/gold interface and the CaF_2/D_2O interface. **b**. Experimental Im$\chi^{(2)}_{yyz}$ and Re$\chi^{(2)}_{yyz}$ spectra in the O-H stretching frequency region at the CaF_2/D_2O interface. **c**. Corresponding phase spectrum

in the O-H stretching frequency region. cps represents counts per second. The grey dashed lines in (**b**) and (**c**) serve as zero lines.

In addition to measuring the D_2O liquid, we also examined the phase of gold by measuring the O-H stretching $\mathrm{Im}\chi^{(2)}_{ssp}$ spectrum of D_2O vapor, where the signal is significantly stronger, as previously adopted by Tahara et al.[17]. In our measurements, the cell was first flowed with D_2O liquid and then purged with D_2O vapor for ~ 1 hour before the measurement to minimize the effect of residual H_2O in the sample cell. The $\mathrm{Im}\chi^{(2)}_{ssp}$ spectrum for the D_2O vapor is shown in Fig. S6. Our data indicate that the O–H stretching region (3000–3700 cm⁻¹) is not necessarily zero for the D_2O vapor measurement due to residual H_2O between the graphene and the CaF_2 substrate, a more reliable phase determination can be made by analyzing the high-frequency region (3700–3800 cm^{-1}), where no resonance response is expected. From this analysis, we infer a phase of 48 degrees for the CaF_2/gold interface, which closely matches the phase obtained for the D_2O liquid when considering the entire O-H stretching frequency region (3000–3700 cm^{-1}), measured at 49 degrees. These results confirm the reliability of our phase measurement method.

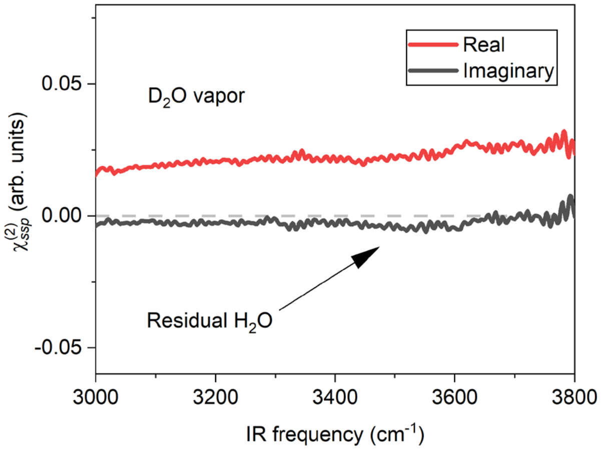

Fig. S6. Phase measurement from D_2O vapor. Experimental $\chi^{(2)}_{ssp}$ spectrum of D_2O vapor. The grey dashed line serves as a zero line.

To validate the accuracy of our Fresnel factor correction method, which assumes a frequency-independent refractive index of gold, we extracted the $\mathrm{Im}\chi^{(2)}_{yyz}$ spectra using both frequency-dependent and frequency-independent refractive indices of gold. The data are very similar (Fig. S7a and S7b). This can be understood by noting that the phase change introduced by the frequency-

dependent refractive index—arising from the LO light reflectivity and Fresnel factor ($r_{\mathrm{LO}}^{*} \times F_{ssp}$)—is only ~1.5° across the studied frequency range (see Fig. S8). This phase shift is smaller than the measurement uncertainty (~3°). It is important to note that our measurements utilize heterodyne detection, meaning the LO light reflectivity (r_{LO}^{*}) is inherently included in the calculations. Interestingly, while the Fresnel factor's amplitude increases monotonically from low to high frequencies—consistent with the findings of Prof. Poul Petersen's group[19]—the inclusion of LO light reflectivity results in a total product ($r_{\mathrm{LO}}^{*} \times F_{ssp}$) that varies by only ~24% across the frequency range. This variation is insignificant to change the lineshape of the $\mathrm{Im}\chi_{yyz}^{(2)}$ spectrum (Fig. S7a). Therefore, we conclude that the frequency dependence of gold's refractive index does not impact our data or conclusions.

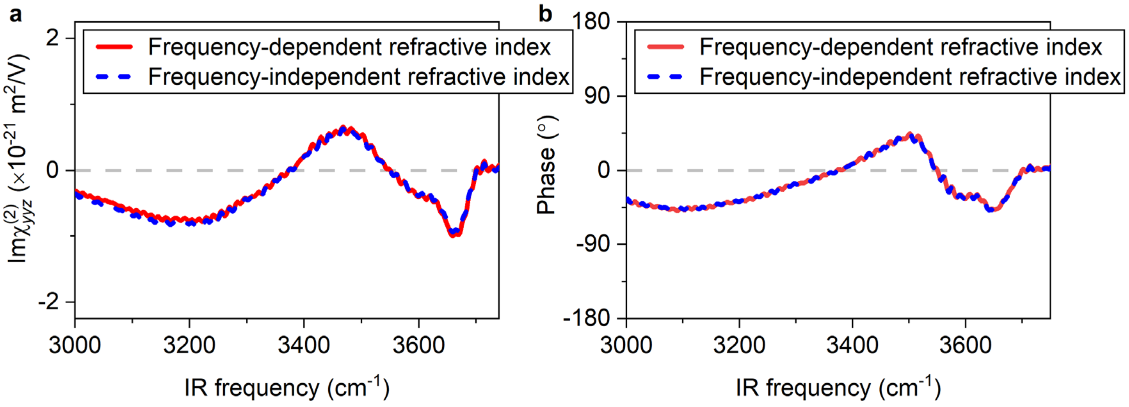

Fig. S7. Effect of refractive index of gold. **a**. $\mathrm{Im}\chi_{yyz}^{(2)}$ spectra of the nanoconfined 3L water, obtained using Fresnel factor correction based on the frequency-dependent and frequency-independent refractive indices of gold in the O-H stretching frequency region. **b**. Corresponding phase spectra. For Fresnel factor correction based on the complex refractive index of gold, the phase was corrected by 136° for comparison. The grey dashed lines in (**a**) and (**b**) serve as zero lines.

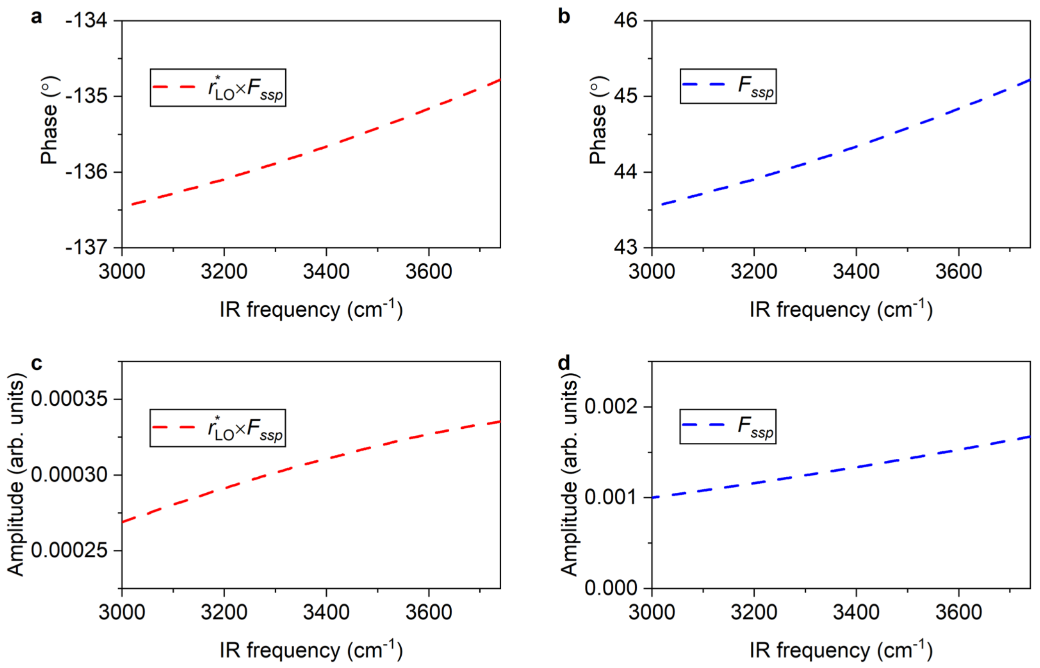

Fig. S8. Phase of gold. a, **b**. Calculated phase of (**a**) $r_{\mathrm{LO}}^{*} \times F_{ssp}$ and (**b**) F_{ssp} for the CaF_2/gold interface in the O-H stretching frequency region. **c**, **d**. Corresponding amplitudes.

10. *Ab Initio* Molecular Dynamics (AIMD) Simulation

We carried out SFG spectra simulations for the CaF_2(111)/water interface, the water/graphene interface, and the nanoconfined water systems using *ab initio* molecular dynamics (AIMD) simulations with the mixed Gaussian and plane wave approach as implemented in the CP2K code[20,21]. We used the (111) facet as the model of the CaF_2 surface because this facet provides the thermodynamically most stable surface[22]. Note that the facet of the CaF_2 sample used in the experiment is unknown, and it tends to be in a polycrystalline phase. This uncertainty may slightly affect the lineshape of the simulated spectra compared to the experimental data, shown in Fig. 2 of the main text. We used the revPBE[23,24] exchange-correlation (XC) functionals together with the empirical van der Waals (vdW) correction scheme of Grimme's D3(0)[25] method. The choice of revPBE+D3 is based on our previous study[26] as it could give reasonable spectral interfacial properties compared with the experimental data. The core electrons were described using the Norm-conserving Goedecker-Teter-Hutter pseudopotentials[27,28]. We employed the short-ranged MOLOPT double-valance ζ basis with one set of polarization functions (DZVP) and the plane wave density cutoff of 400 Ry. The time step for integrating the equation of motion was set to 0.5 fs. All simulations were performed at 300 K in the NVT ensemble with the thermostat of the

canonical sampling through the velocity rescaling method[29]. The surface dipole correction was used to remove the impact of the macroscopic dipole in the image cell[30].

We ran AIMD simulations for the three nanoconfined systems with different fractions of water, the CaF_2/aqueous LiCl solution, and the aqueous LiCl solution/graphene interfaces (Fig. S9). For all the systems, we used the same simulation cell, where the cell vectors were $\vec{a} = (14.76\ \text{Å}, 0\ \text{Å}, 0\ \text{Å})$, $\vec{b} = (7.38\ \text{Å}, 12.78\ \text{Å}, 0\ \text{Å})$, and $\vec{c} = (0\ \text{Å}, 0\ \text{Å}, 50\ \text{Å})$. $\vec{a}$ and $\vec{b}$ formed the surface, while $\vec{c}$ formed the surface normal. This surface unit cell ($\vec{a}$ and $\vec{b}$) corresponds to the graphene-*p*(6×6) hexagonal cell. The lattice mismatch between the graphene sheet and the $CaF_2(111)$ surface was compensated by compressing the $CaF_2(111)$-*p*(4×4) by 4.5%. The CaF_2 consisted of three layers (one layer is composed of 16 formula units of CaF_2), while the graphene sheet was composed of 72 carbon atoms.

The positive charge of the CaF_2 surface originates from the dissolution of fluoride ions.[31] To mimic charging of the $CaF_2(111)$ in our simulations, we detached one fluorine atom from the topmost surface of CaF_2 in contact with water according to $\equiv (\mathrm{CaF_2})_X \rightleftharpoons \equiv (\mathrm{CaF_2})_{X-1} + \equiv (\mathrm{CaF})^+ + \mathrm{F^-(aq)}$[32]. The detached fluoride ion was solvated in water to maintain the charge neutrality of the total system, required for converging the electrostatic energy. Given the small cell size in the AIMD simulation, the detachment of one fluoride ion from the CaF_2 surface leads to a charge density of ~98 mC/m^2, larger than the experimentally estimated surface charge (40±10 mC/m^2, see Supplementary Discussion S1 for more details). The AIMD simulation is used to train a neural network to infer force fields for the machine-learning assisted MD (MLMD) simulations. In the MLMD simulation, we can increase the cell size and reduce the surface charge density, since the computational cost is much reduced compared to the AIMD simulation. As such, we can perform the MLMD simulation with the surface charge density of 49 mC/m^2. We noted that such a similar protocol is also used in e.g. Ref.[33].

We prepared the three samples for the nanoconfined water systems. The sample containing the smallest fraction of water (the thickness of the water layer: ~8 Å) was composed of 49 H_2O molecules and 2 pairs of Li^+ and Cl^- ions, the sample containing the second smallest fraction of water (the thickness of water layer: ~12 Å) was composed of 77 H_2O molecules and 3 pairs of Li^+ and Cl^- ions, and the sample containing the largest fraction of water (the thickness of water layer: ~16 Å) was composed of 99 H_2O molecules and 4 pairs of Li^+ and Cl^- ions. The resulting LiCl

concentrations for the three nanoconfined water systems were around 2 M. For the CaF_2/water sample, we contained 159 H_2O molecules and 3 pairs of Li^+ and Cl^- ions. The resulting LiCl concentration was ~1 M. For the water/graphene sample, we contained 136 H_2O molecules without adding ions because the water spectrum at the water/graphene interface is insensitive to ions. Furthermore, we carried out the simulation without Li^+ and Cl^- ions at the CaF_2/water interface.

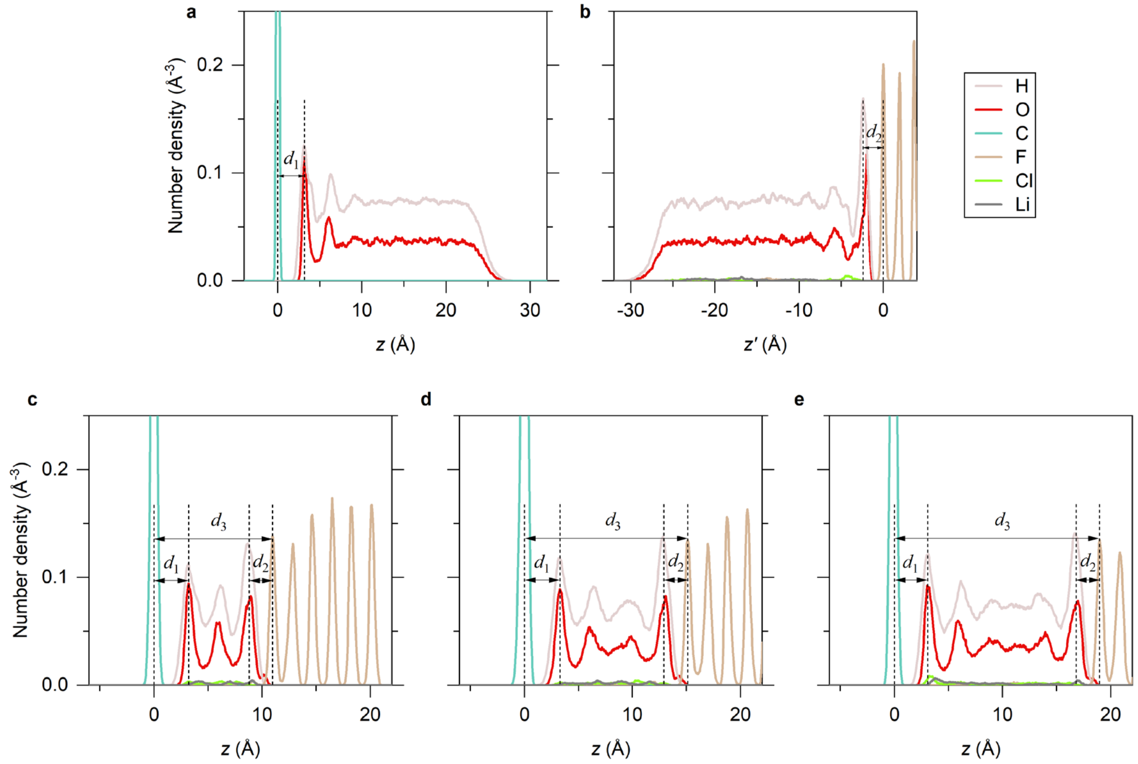

Fig. S9. The axial profiles of the number density along the surface normal axis for (a) the water/graphene interface, (b) the CaF_2/water interface system as well as (c-e) the nanoconfined systems with a water thickness of (c) ~8 Å, (d) ~12 Å, and (e) ~16 Å. The origin points of the z-axis and z'-axis were set to the position of the graphene sheet (**a**, **c**, **d**, **e**) and the position of the first layer of F atom (**b**) in the cell, respectively. The obtained distances between the topmost water layers and the graphene sheet are (**a**) d_1 = 3.3 ± 0.1 Å, (**c**) d_1 = 3.2 ± 0.1 Å, (**d**) d_1 = 3.3 ± 0.1 Å, (**e**) d_1 = 3.2 ± 0.1 Å. The distances between the water slab and CaF_2 are (**b**) d_2 = 2.3 ± 0.1 Å, (**c**) d_2 = 2.3 ± 0.1 Å, (**d**) d_2 = 2.3 ± 0.1 Å, (**e**) d_2 = 2.3 ± 0.1 Å. The distances between the graphene and CaF_2 in the confined water systems are (**c**) d_3 = 11.0 ± 0.1 Å, (**d**) d_3 = 15.2 ± 0.1 Å, (**e**) d_3 = 19.2 ± 0.1 Å.

Note that in this simulation, a fluoride is solvated in the water, to achieve the charge neutrality of the system. For all the systems, the resulting thickness of the vacuum regions in the simulation box was >15 Å (Fig. S9). As a result, the other side of the interfaces for the CaF_2/water and water/graphene samples, which are generated in the slab geometry is the water/air interface. We generated 5 independent configurations for each sample. We equilibrated the systems by running 5 ps, and we sampled the >30 ps trajectories, from which we computed the SFG spectra and performed the analysis.

11. Machine Learning Force Field MD Simulation

The surface charge of the CaF_2/water interface for the CaF_2 substrate employed in our SFG measurements was experimentally estimated to be 40±10 mC/m^2 at a neutral pH condition (see Supplementary Discussion S1). This value is smaller than the surface charge density used in the AIMD simulation; to simulate the CaF_2/water interface and the nanoconfined water system with the experimentally estimated surface charge density, one needs to expand the simulation cell size. However, due to the huge computational cost of the AIMD simulation, it is highly challenging to scale up the system size. To overcome the difficulty, we used the machine learning force field (MLFF) based molecular dynamics simulation technique.

We constructed a deep potential model to fit the potential energy surface (PES) which is implemented in the DeePMD-kit package[34–36] for the CaF_2/water system as well as the nanoconfined water system. We used the "se_e2_a" descriptor model with local environment cutoff and smooth cutoff parameters set as 6.0 and 0.5 Å. The embedding bet was composed of the hidden layers with their sizes of (25, 50, 100), and the fitting net had the hidden layers with their size of (240, 240, 240). We constructed 4 MLFF models using a concurrent learning workflow implemented in the DP-GEN package[37]. We randomly selected 2800 structures from the AIMD simulated data to train 4 initial models. We repeated the "exploration", "labelling", and "training" process for the large cells explained below until the deviations among the MLFF models were below 0.2 eV/Å for 99% of structures in the sampling trajectories. After that, a final model was trained based on all the training data. The resulting FF model vs *ab initio* data are shown in Fig. S10 and S11. Note that, we did not employ the DeePMD with long-range corrections to fit the PES, because the calculated spectra without the long-range corrections show a good agreement with experimental data with current ions concentration. (see Supplementary Discussion S14).

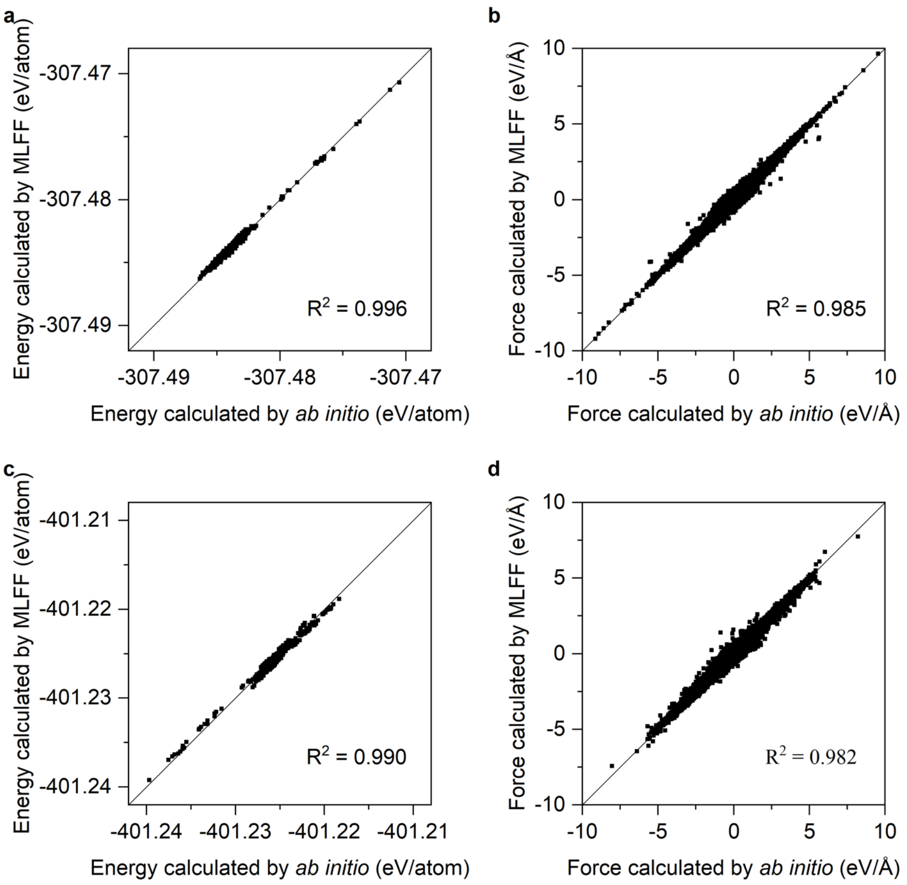

Fig. S10. Comparisons of energies and forces obtained by *ab initio* method and MLFF on the test data set of CaF_2/water (a-b) and nanoconfined water system (c-d). The coefficient of determination (R^2) is shown in each plot as well as the RMSE for energy and forces.

We constructed the cell composed of $2\vec{a}$, $2\vec{b}$, and $\vec{c}$, where we detached two fluorine atoms from the CaF_2 surface and made the fluorine ions solvated in water. This led to a surface charge density of 49 mC/m^2. This surface charge value is consistent with the experimentally obtained value (40±10 mC/m^2). For the CaF_2/water sample, we contained 596 H_2O molecules and 12 pairs of Li^+ and Cl^- ions. For the nanoconfined water sample, we contained 196 H_2O molecules and 8 pairs of Li^+ and Cl^- ions. The nanoconfined water system provides a water layer thickness of ~8.0 Å, close to the AFM data of ~8.2 Å. We also constructed another nanoconfined water sample using a higher ion concentration with the same cell size, which contained 172 H_2O molecules and 20 pairs of Li^+ and Cl^- ions. This combination increases the LiCl concentration thrice, from ~2 M to ~6 M.

After generating the MLFF model, we carried out the force field MD simulation for CaF_2/water and nanoconfined water systems of high and low LiCl concentrations by using the LAMMPS package[38]. The equation of motion was integrated with a timestep of 0.5 fs. We used the Nose-Hoover thermostat with a temperature damping parameter of 1 ps. We ran the MLFF-MD simulation with NVT ensemble for the total 5 independent systems at 300 K for low LiCl concentration with total trajectories length of >2 ns. and a total of four independent systems at 300 K for high concentration with total trajectories length of 4 ns. From all the MD trajectories, we calculated the SFG spectra and performed the analysis.

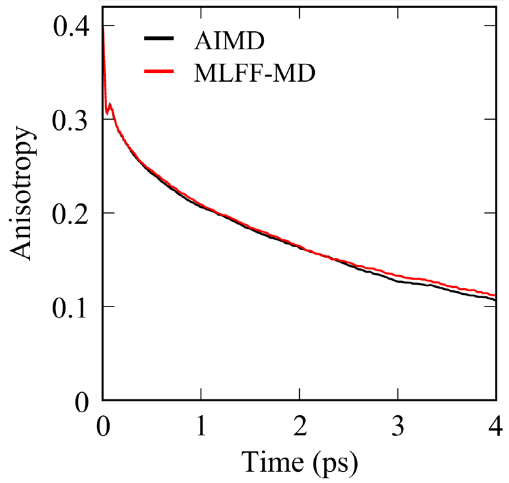

Fig. S11. Comparisons of dynamic property (anisotropy decay time of the free OH of the water molecules near the CaF_2/water interface) obtained by ab initio method and MLFF on the test dataset of CaF_2/water.

12. $\chi^{(2)}$ Spectrum Calculation

We computed the SFG spectra by using the AIMD or MLFF MD trajectories by using the surface-specific velocity-velocity auto-correlation function (ssVVAF) approach[39]. This method enables one to compute the SFG spectra with a reasonable s/n ratio solely from the MD trajectories. Within this ssVVAF formalism, the resonant part of the SFG susceptibility, $\chi_{xxz}^{(2),R}(\omega)$, can be given as:

$$\chi_{xxz}^{(2),R}(\omega) = \frac{Q(\omega)\mu'(\omega)\alpha'(\omega)}{i\omega^2}\chi_{xxz}^{ssVVAF}(\omega), \tag{S5}$$

$$\chi_{xxz}^{ssVVAF}(\omega) = \int_0^\infty dt e^{-i\omega t} \langle \sum_i g_{ds}\big(z_i(0)\big)\dot{r}_{z,i}^{OH}(0)\frac{\dot{\vec{r}}_i^{OH}(t)\cdot\vec{r}_i^{OH}(t)}{\left|\vec{r}_i^{OH}(t)\right|}\rangle, \tag{S6}$$

where $g_{ds}(z_i)$ is the truncation function for the dividing surface to selectively extract the vibrational responses of water molecules near the interface:

$$g_{ds}(z_i) = \begin{cases} 0 \text{ for } z_i \geq z_{\text{ds}} \\ 1 \text{ for } z_i < z_{\text{ds}} \end{cases}, \tag{S7}$$

where z_{ds} is the z-coordinate of the dividing surface and z_i is the z-coordinate of the O atom of the ith O-H bond. The z_{ds} value was set to decouple the responses of the CaF_2/water interface and graphene/water interface from the water/air interface. Note that for the confined water system, we did not use any dividing surface; the SFG response was calculated using the whole water slab. We set the origin point as the averaged position of the first layer of F atoms for the CaF_2/water interface, and the averaged position of C atoms for the graphene/water interface. The z_{ds} value is set to 12 Å for both the CaF_2/water system and the graphene/water system. Due to the geometry, the $g_{ds}(z_i)$ function for CaF_2/water system is slightly modified as:

$$g_{ds}(z_i) = \begin{cases} 0 \text{ for } z_i \leq -z_{\text{ds}} \\ 1 \text{ for } z_i > -z_{\text{ds}} \end{cases}. \tag{S8}$$

The frequency-dependent induced transition dipole moment and polarizability due to the solvation effects were included by using the frequency-dependent transition dipole moment ($\mu'(\omega)$) and polarizability ($\alpha'(\omega)$)[40,41]:

$$\mu'(\omega) \equiv \left(1.377 + \frac{53.03(3737.0 - \omega)}{6932.2}\right)\mu^0, \tag{S9}$$

$$\alpha'(\omega) \equiv \left(1.271 + \frac{5.287(3737.0 - \omega)}{6932.2}\right)\alpha^0, \tag{S10}$$

where ω is in cm^{-1}. μ^0 and α^0 are permanent dipole moments and permanent polarizability of OH chromophores, respectively. $Q(\omega)$ is the quantum correction factor given by[42]:

$$Q(\omega) = \frac{\beta\hbar\omega}{1 - \exp(-\beta\hbar\omega)}, \tag{S11}$$

where $\beta = 1/kT$ is the inverse temperature.

We calculated the SFG response at the water/air interface for both the CaF_2/water and water/graphene systems, to verify the accuracy of our calculations. In Fig. S12, we present the simulated SFG spectra for the water/air interface from these two sample configurations. As shown, the spectra are indistinguishable, further validating the accuracy of our simulation results.

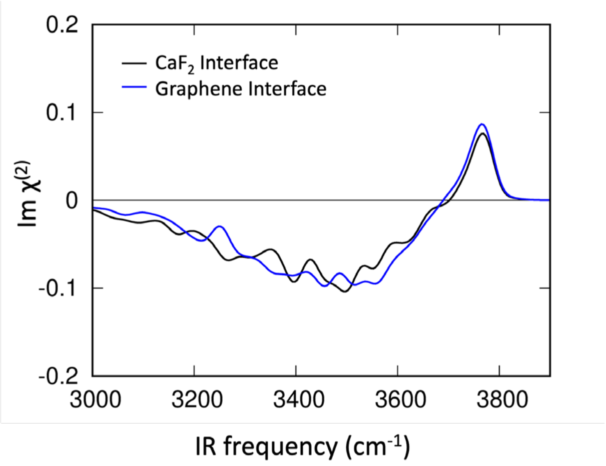

Fig. S12. Simulated Water/Air interface SFG spectra. The simulated SFG spectra of the water/air interfaces based on the CaF_2/water and water/graphene samples.

Because the CaF_2/water systems contained both the CaF_2/water interface and the water/air interface, we needed to exclude the contribution from the water/air interface. To this end, we computed the layer-by-layer SFG contribution by including the interfacial water layers near the CaF_2/water interface by using the AIMD data. The results shown in Fig. S13 indicate that the $z_{\rm ds}$ value is set to 12 Å for the CaF_2/water interface is sufficient to exclude the contribution from the water/air interface.

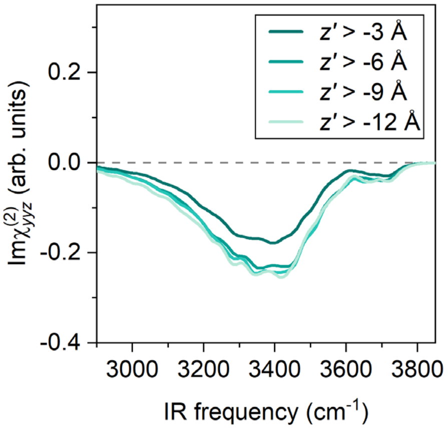

Fig. S13. Layer-by-layer contribution of SFG spectra at the CaF_2/water interface obtained by using AIMD data.

This notion was further supported by the dipole orientation of the water molecules (Fig. S14). Even without any Li^+ and Cl^- in water and only with an F^-, the orientation is saturated at z>-12 Å. These

data guaranteed that the inclusion of the water molecule in the z>-12 Å region is sufficient to reproduce the SFG signal.

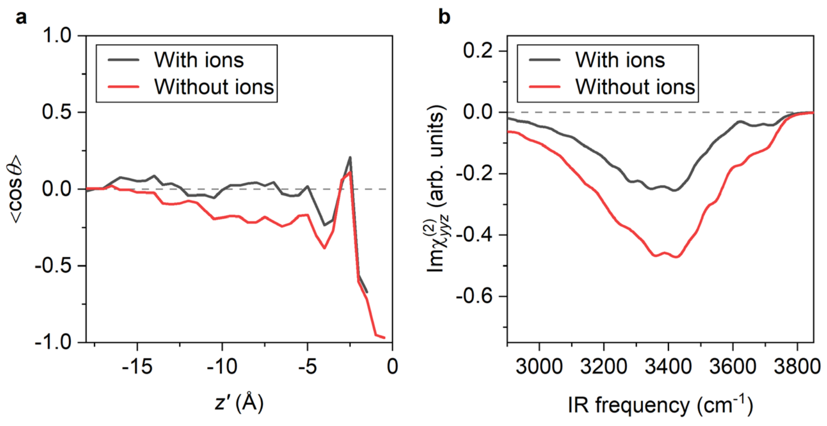

Fig. S14. Comparison of (a) dipole orientation and (b) AIMD based Im$\chi^{(2)}_{yyz}$ spectra of CaF_2/water interfaces with and without Li^+ and Cl^- ions.

Supplementary Discussion

S1. LiCl Concentration and Its Effect on SFG Spectra

At a charged interface, an electric field penetrates into the bulk solution and induces alignment and polarization of water molecules in the diffuse layer, providing a bulk contribution to the SFG signal[43–45]. Solvated ion screens the electric field generated at the charged surface, whose concentration, therefore, significantly affects the SFG signal. Such ion concentration dependence is vanishingly weak at high ion concentrations (> 1 M, for example), according to the Gouy-Chapman theory[17,45,46]. Nevertheless, recent studies proposed that ions of high concentrations could also alter the water structure near the charged surface, affecting the SFG spectra, such as at the SiO_2 surface[17]. Here, we examined the sensitivity of the SFG spectra to the LiCl concentration for the CaF_2/water interface as well as the nanoconfined water system.

First, we measured $\mathrm{Im}\chi^{(2)}_{yyz,\,\mathrm{CaF_2/W}}$ spectra at the CaF_2/water interface by varying the LiCl concentrations. The data are displayed in Fig. S15a. Only a minor reduction of the SFG amplitude was observed when increasing the LiCl concentration from 1 M to 8 M at the CaF_2/water interface. This can be fully explained by the screening of the surface charge of the CaF_2 upon increasing the ion concentration, which is effectively completed when the electrolyte concentration reaches 1 M. Indeed, after removing the bulk contribution ($\chi^{(3)}$) by considering a constant surface charge density (σ_0) within the Gouy-Chapman theory (Eq. S12)[43–45], the surface contribution ($\chi^{(2)}_{yyz,\,\mathrm{s}}$) exhibits negligible change in both peak intensity and lineshape, confirming that σ_0 is constant (Fig. S15b). This indicates that the ions do not (or negligibly) induce further surface charging (*i.e.*, change of the density of surface charges) of the CaF_2 surface. The negligible surface charging of CaF_2 allows us to directly verify the validity of Eq. (1) in our main text and is one of the main reasons we chose the CaF_2 substrate to construct the two-dimensional nanoconfined water system.

$$\chi^{(2)}(\sigma_0, c) = \chi^{(2)}_{\mathrm{s}} + \chi^{(3)}\phi_0(\sigma_0, c)\,\kappa(c)/(\kappa(c) - i\Delta k_z)\,, \qquad \text{(S12)}$$

where $\chi^{(3)}$ represents the third-order nonlinear susceptibility originating from bulk water, ϕ_0 is the electrostatic potential, κ the inverse of Debye screening length, *c* electrolyte concentration, and Δk_z the phase-mismatch of the SF, visible, and IR beams in the depth direction.

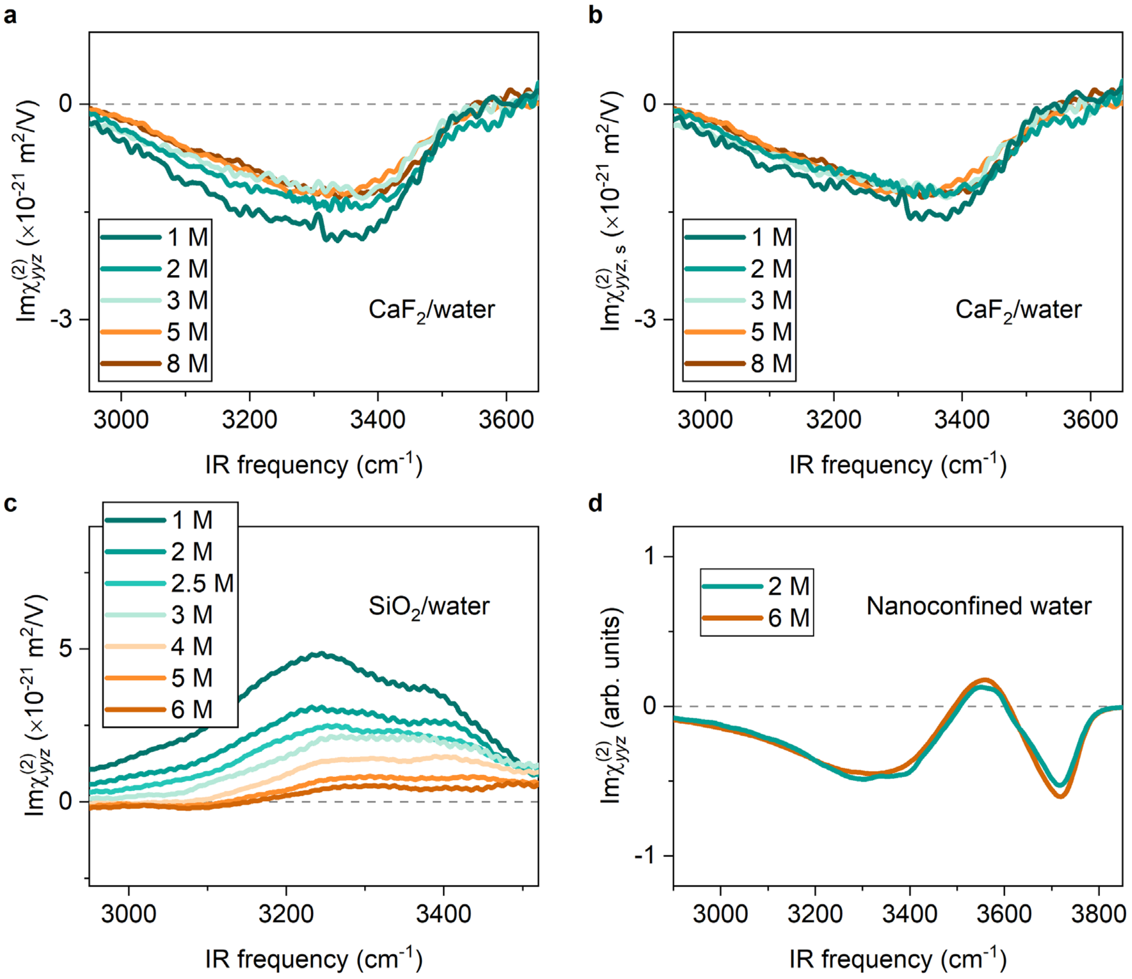

Fig. S15. Effect of LiCl concentration. a, **c**. The $\mathrm{Im}\chi^{(2)}_{yyz}$ spectrum at the (**a**) CaF_2/bulk water interface and (**c**) SiO_2/bulk water interface at different LiCl concentrations. **b**. The $\mathrm{Im}\chi^{(2)}_{yyz,\,\mathrm{s}}$ spectrum at the CaF_2/bulk water interface at different LiCl concentrations. **d**. Simulated SFG spectra with different ion concentrations for the ~8 Å nanoconfined system computed from MLFF-MD data. The dashed lines in (**a-d**) serve as the zero lines.

Unlike the CaF_2 surface, SFG signals at the SiO_2 surface are sensitive to the ion concentration, probably because of the ion-induced further surface charging[17]. To confirm this, we measured $\mathrm{Im}\chi^{(2)}_{yyz,\,\mathrm{SiO_2/W}}$ spectra at the SiO_2/water interface by varying the LiCl concentrations. The data are displayed in Fig. S15c. Consistent with Ref.[17], the $\mathrm{Im}\chi^{(2)}_{yyz,\,\mathrm{SiO_2/W}}$ spectra vary significantly with the high ion concentration, implying that ion-induced surface charging occurs at the SiO_2/water interface. Remarkably, surface charging of the SiO_2 allows us to estimate the ion concentration of

the confined LiCl solution by comparing the confined water signal with the sum signal of signals at the bulk water/graphene interface and the SiO_2/bulk water interface at different LiCl concentrations. The data is shown in Fig. S16. The SFG signal of water confined between graphene and SiO_2 substrate matches the sum signal of the water/graphene interface and SiO_2/water interface signals at a LiCl concentration of ~2-3 M. Assuming that the SiO_2 undergoes the same ion-induced charging behavior in nanoconfined water system and SiO_2/bulk water system, we conclude that the LiCl concentration of the nanoconfined water in such two-dimensional nanoconfined water system is ~2-3 M.

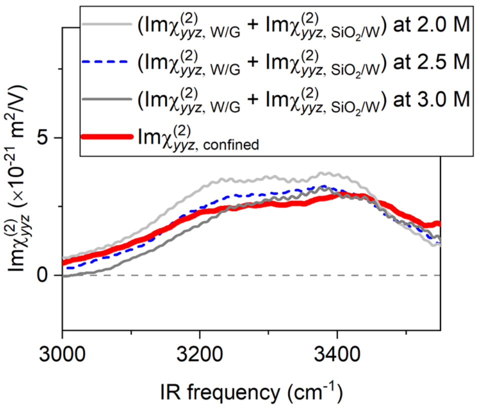

Fig. S16. Estimation of ion concentration under confinement and the effect of the supporting substrate. Experimental $\mathrm{Im}\chi^{(2)}_{yyz}$ spectra of the nanoconfined water between graphene and SiO_2. The sum of the water/graphene and SiO_2/water signals obtained using different concentrations of LiCl is also shown for comparison. The grey dashed line serves as zero line.

We further examined the LiCl concentration dependence of the SFG spectra for the nanoconfined system. Here, we employed the machine learning MD simulations to examine this, because our sample preparation method could control the LiCl concentration only passively. The calculated SFG spectra with 2 M and 6 M LiCl are shown in Fig. S15d. We used the nanoconfined system with a thickness of 8 Å. The SFG spectra are insensitive to ion concentration even in the nanoconfined system. Furthermore, we also computed the dipole orientation profiles. The data shown in Fig. S17 indicate that the structure of the central water layer remains largely unaffected by the high-concentration LiCl.

We also examined the effect of ions. To this end, we prepared the nanoconfined water using KCl instead of LiCl and measured the $\mathrm{Im}\chi^{(2)}_{yyz}$ spectra. The data shown in Fig. S18 suggest that the SFG spectra are insensitive to ion type.

In contact with water (at neutral pH), the CaF_2 surface is positively charged[8,47]. Here, we estimated the surface charge density (σ_0) of the CaF_2 substrate. To this end, we measured the SFG signals at the CaF_2/water interface at different ion concentrations ($c_1 = 10$ mM and $c_2 = 2$ M) and then estimated the σ_0 from the differential spectrum $\Delta\chi^{(2)} = \chi^{(2)}(\sigma_0, c_1) - \chi^{(2)}(\sigma_0, c_2)$ within the Gouy-Chapman theory[8,43–45]. The $\mathrm{Im}\Delta\chi^{(2)}$ spectrum is shown in Fig. S19 and the corresponding experimentally estimated σ_0 is ~40±10 mC/m^2.

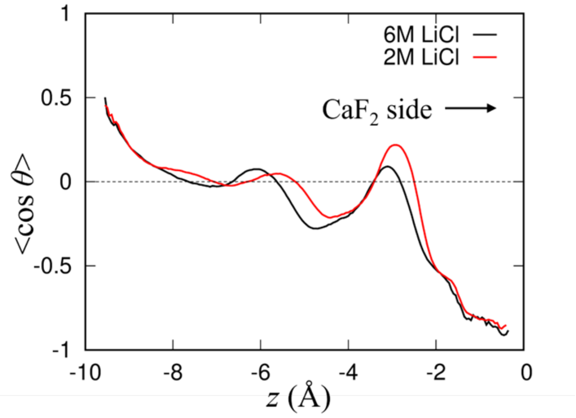

Fig. S17. Depth profiles of the dipole orientation of water for the nanoconfined 3L water sample at different LiCl concentrations, computed by using MLFF-MD data. The angle θ is defined as the angle between the bisector of a water molecule and the surface normal (*z*- or *z*'-axis).

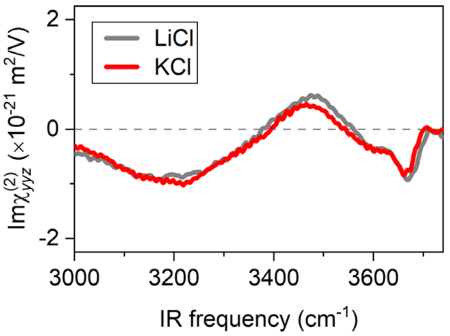

Fig. S18. Ion effect. The Im$\chi^{(2)}_{yyz}$ spectrum of the nanoconfined water containing LiCl and KCl. The dashed line serves as a zero line.

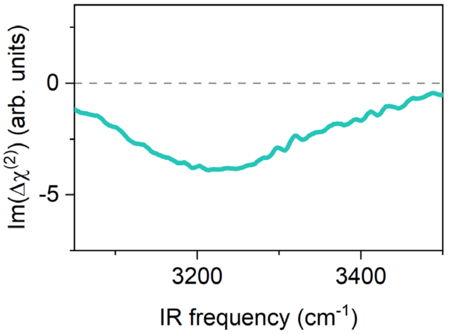

Fig. S19. Im$\Delta\chi^{(2)}$ spectrum for estimation of σ_0 of the CaF_2 substrate. Corresponding σ_0 were estimated following the recipe reported in Ref.[8]. The dashed line serves as a zero line.

S2. Characterization of the CaF_2 Surface

To confirm the CaF_2 surface is flat and uniform within the HD-SFG probe region (the diameter of the laser spot is around 100 μm), we conducted AFM measurements on the bare CaF_2 substrate across a randomly chosen 100×100 μm^2 region. The AFM data shows that while some atomic terraces or defects are present (diagonal lines), the CaF_2 surface appears atomically flat within the SFG probed region, showing an RMS surface roughness (R_q) measuring around 1.3 Å (Fig. S20a). The atomically flat CaF_2 surface is further confirmed by the close-up AFM image (0.5×0.5 μm^2) showing a R_q~1.0 Å comparable to that of a graphite flake (R_q~0.8 Å, Figs. S20b, and c). The graphite flake is a good reference since it is a commonly used atomically flat substrate in nanofluidic devices[3,48]. Importantly, the surface roughness is appreciably smaller than a monolayer of water (~3.7 Å)[1]. We emphasize that, despite using organic solvents for cleaning the surface, the CaF_2 surface is clean as seen from the absence of C-H stretch peaks (2850-2950 cm^{-1}) from possible organic contamination in the SFG spectrum of the nanoconfined water sample (Fig. S20d).

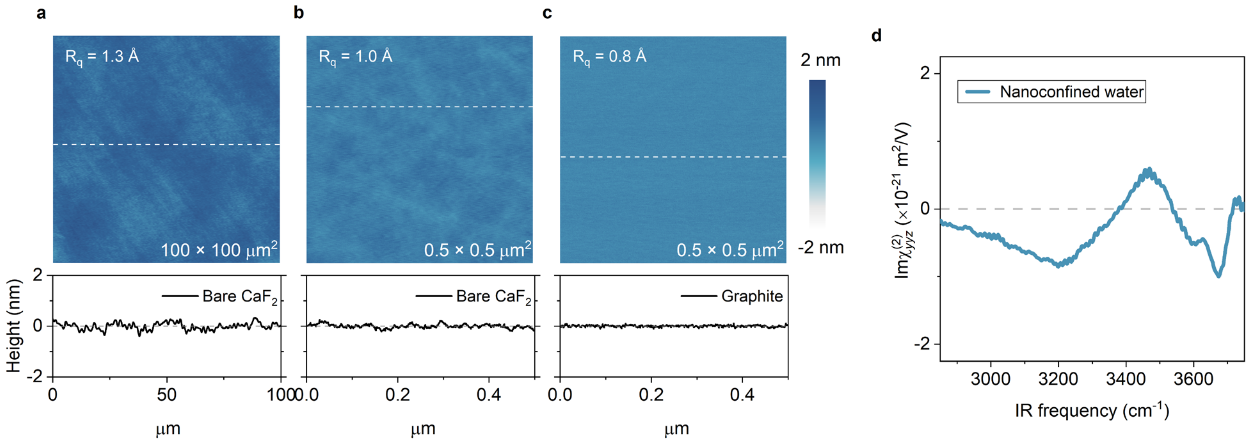

Fig. S20. Characterization of the CaF_2 surface. a. AFM height images of the bare CaF_2 substrate across a 100×100 μm^2 region. **b**, **c**. High-resolution height images of the atomically flat CaF_2 substrate and graphite. The bottom panel in each AFM height image shows the typical height profiles along the white dashed lines in the corresponding AFM height image. The dashed grey lines in the height profiles indicate zero lines. R_q values were calculated across the whole scan area. **d**. Cleanliness of the CaF_2 substrate. The $\mathrm{Im}\chi^{(2)}_{yyz}$ spectrum of the nanoconfined water sandwiched between the graphene sheet and the atomically flat CaF_2 substrate. The dashed line serves as a zero line.

S3. AFM Characterization of the Sample Height

To ensure SFG and AFM probed the same position of the sample, we marked a region at the edge of the sample by covering the unprobed region with gold. An optical image of the sample is presented in Fig. S21a, while Fig. S21b provides a close-up optical image. AFM and SFG measurements were conducted within this marked sample region (with a sample size of ~250×200 μm^2 as seen in Fig. S21b). An optical microscope can identify the nanoconfined water region probed with AFM. In the HD-SFG measurement, we identified the marked region by sliding the sample with a translation stage (~10 μm translation accuracy) and checking the HD-SFG signals; in the gold region, the SFG signal from gold is very strong, and in the bare CaF_2 region, there is negligible water signal. As such, we could identify the nanoconfined water region by minimizing the signal along the *y*-axis and maximizing the water signal along the *x*-axis, where *x*- and *y*-axes are defined in Fig. S21b. The AFM data measured from the marked region is shown in Fig. S21c. Across the 100×100 μm^2 scan region, although some graphene wrinkles appear, the nanoconfined water spreads flat and uniform on the CaF_2 substrate. The height of the graphene together with the nanoconfined water is ~11.5 Å (Fig. S21d). Given that the thickness of the exclusive volume of the graphene sheet is 3.3 Å, the thickness of the nanoconfined water (h) is ~8.2 Å.

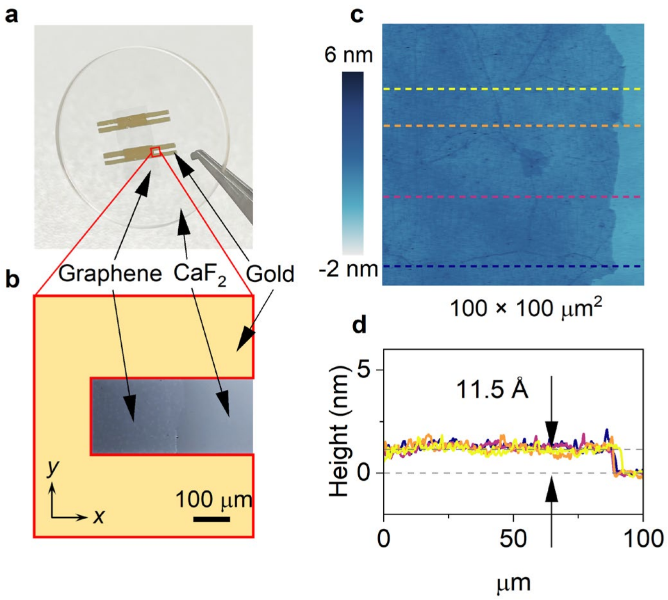

Fig. S21. AFM characterization of the nanoconfined water. a. An optical image of the nanoconfined water sample with a gold marker. **b**. A close-up optical image of the nanoconfined water sample surrounded by gold. **c**. AFM height image of the nanoconfined water sample. **d**. The height profiles along the corresponding-colored dotted lines in (**c**).

S4. Raman Characterization of the Graphene Sheet

Confinement of water molecules between the graphene and the CaF_2 substrate is supposed to induce changes in the strain (ε) and charge density (n) of the graphene. The changes in ε and n will cause the frequency shift of the Raman *G*-band (ω_G, ~1582 cm^{-1}) and *2D*-band (ω_{2D}, ~2678 cm^{-1})[49]. Accordingly, to probe the effect of confined water on the graphene sheet, we measured the Raman *G*-band and *2D*-band. A typical Raman data is shown in Fig. S22a and the *G*-band and *2D*-band data collected at a randomly selected region are shown in Fig. S22b. A slight blue-shift of ω_G and ω_{2D} is observed compared to the graphene supported on a CaF_2 substrate without water molecules being confined.

As increasing n (ε) of an intrinsic graphene, its values of (ω_G, ω_{2D}) will move from *O* along v_H (v_S) as shown in Fig. S22b, where v_H (v_S) represents the unit vector for n (ε) in the ω_G-ω_{2D} vector space. ε and n on the graphene can be independently determined[50,51] through correlation analysis of the frequency shift of ω_G and ω_{2D}. The variations in ω_G and ω_{2D} of the CVD graphene on the CaF_2 substrate are purely induced by strain with negligible charge doping ($n < 10^{12}$ cm^{-2}). Most graphene experiences a compressive strain varying from 0 to 0.05% due to the presence of wrinkles in CVD graphene. The situation becomes different when water molecules are confined between the graphene monolayer and the CaF_2 substrate. In this case, in most regions, graphene experiences a tensile strain ranging from 0 to 0.05% owing to the successful confinement of water molecules. Such a tensile strain enables us to approximately determine the vdW pressure experienced by the trapped solution to be 0 to 500 MPa[52]. Besides the variation along the v_s, most data points move towards v_h, indicating a slight hole doping ($n^{hole} \sim 10^{12}$ cm^{-2}). The Raman spectral analysis of the graphene also supports the successful confinement of water molecules between the graphene and the CaF_2 substrate.

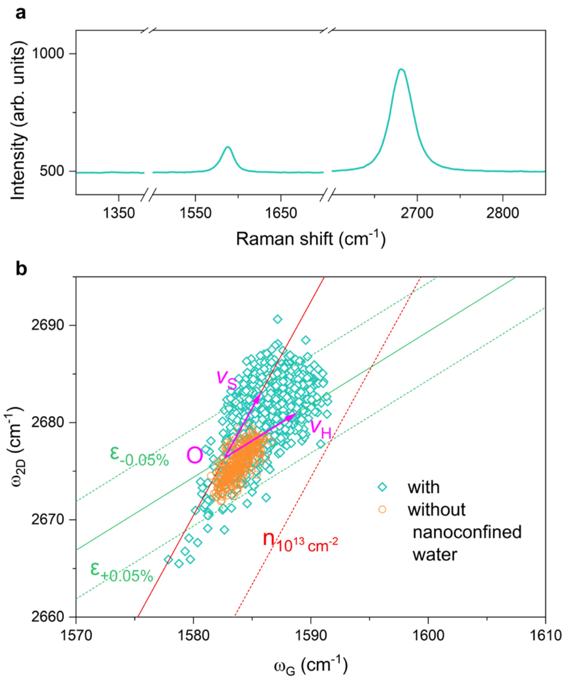

Fig. S22. Raman characterization of the graphene sample. a. Raman spectrum of the graphene layer at a randomly selected region. **b**. Correlation analysis of the frequency shift of ω_{G} and ω_{2D}. The red and green lines represent the correlation induced by pure strain and hole doping effects, respectively. Point *O* represents the intrinsic frequencies of the *G*- and *2D*-bands that are not affected by strain or charges and are 1,581.8 cm^{-1} and 2,676.9 cm^{-1}, respectively[49]. The averaged strain-sensitivity factor *G*-band is − 69.1 ± 3.4 $cm^{-1}/\%$[53]. The positive charge-sensitivity factor of the *G*-band is adopted from a reference (red dashed line)[49].

S5. Capillary Condensation

Our two-dimensional nanoconfined water system, utilizing a graphene sheet and a flat CaF_2 substrate, was based on capillary condensation[3], which can be tuned through well-established methods by varying relative humidity (RH)[3,54,55]. We varied RH by purging the cell with N_2 of different RHs. The RH of the N_2 was tuned by changing the relative flow rate of a mixture comprising dry N_2 and wet N_2 while maintaining the overall N_2 flow rate into the sample cell. To guarantee the RH control, a microscopic humidity sensor was connected to the sample cell near the cell outlet to measure the RH in the sample cell (Fig. S3).

To show the tunable h by varying RH, we conducted AFM measurements under controlled RH. The data measured across the 100×100 μm^2 region are shown in Fig. S23a. Decreases the RH from ~25% to ~5%, h decreases from ~8 Å to below 3 Å gradually over approximately 6 hours (Fig. S23b). Importantly, the variation of h with RH can be accurately modeled by the Kelvin equation (Eq. S13, see Fig. S23c), confirming that the effective confinement of water between the graphene sheet and the CaF_2 substrate predominantly arises from capillary condensation[3,54,56]. In our measurements, we limited the RH in the low range (~40% to ~5%) to avoid bulk water absorption at the graphene/air interface.

$$h = \frac{-2\sigma \cos\theta}{k_B T \rho_N \ln(\mathrm{RH})}, \tag{S13}$$

where σ is the surface tension of water at room temperature T[57], k_B is the Boltzmann constant and ρ_N is the number density of water, θ is the contact angle of water on the walls' material. This equation has been found to hold tor both strongly hydrophilic (such as mica) and weakly hydrophilic (like graphite) capillaries, even when subjected to atomic-scale confinement[3]. The effective water contact angle of a CaF_2/graphene system is in the range of 40° to 60°[58]. Remarkably, within experimental uncertainty, the calculated h as a function of RH using Eq. S13 provides quantitative agreement with the experimental data, underscoring that capillary condensation predominantly accounts for the successful confinement of water.

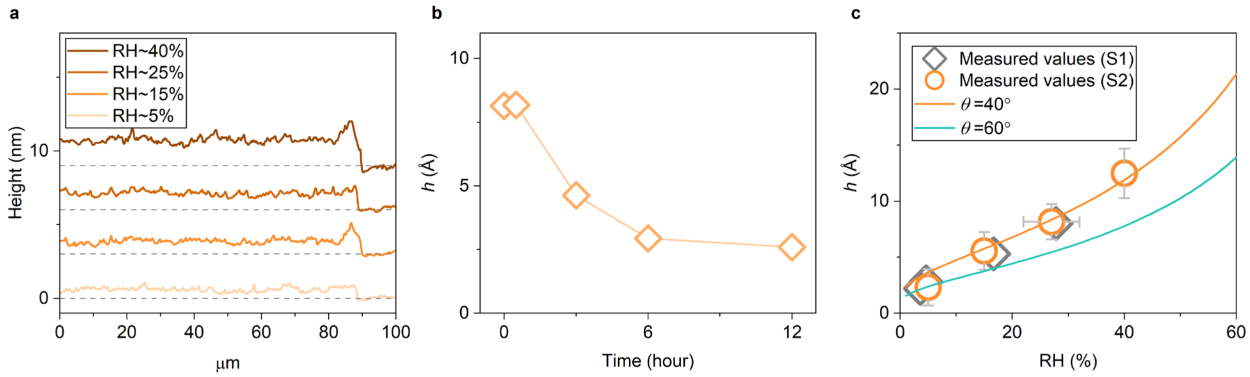

Fig. S23. Tuning the thickness of the nanoconfined water. a. AFM height profiles probing the graphene edge at different RHs. For each RH, the sample was allowed to equilibrate for more than 6 hours before measurements. The data are offset by 3 nm for clarity and the dashed lines serve as zero lines. **b**. h as a function of the time upon the RH change from 25% to 5%. **c**. h as a function of the RH. The lines show the predictions by the Kelvin equation for two CaF_2/graphene water contact angles. In (**b**) and (**c**), h was calculated from the AFM height profiles assuming the graphene thickness of 3.3 Å.

S6. SFG Spectra at *ppp* Polarization

As a comparison, we measured SFG signal ($\chi^{(2)}_{ppp,\,\mathrm{eff}}$) at *ppp* polarization combination for the nanoconfined 3L water. The data are shown in Fig. S24. In the measured $\chi^{(2)}_{ppp,\,\mathrm{eff}}$ signal, of particular interest is the $\chi^{(2)}_{zzz}$ component which, in principle, can be inferred from the measured $\chi^{(2)}_{ppp,\,\mathrm{eff}}$ and $\chi^{(2)}_{ssp,\,\mathrm{eff}}$ signals through Fresnel factor correction assuming a specific interfacial dielectric constant model[59–61]. Nevertheless, as noted in previous studies[60,62], the generally employed homogeneous interfacial dielectric model, either the Lorentz model or Slab model, are insufficient to provide accurate correction for the *ppp* data. Consequently, directly comparing the amplitudes of $\chi^{(2)}_{zzz}$ signals for nanoconfined water and interfacial water remains challenging and is beyond the scope of this study. For reasons mentioned above, our discussion primarily focuses on the *ssp* polarization combination signal ($\chi^{(2)}_{ssp}$), since it allows for straightforward spectral interpretation.

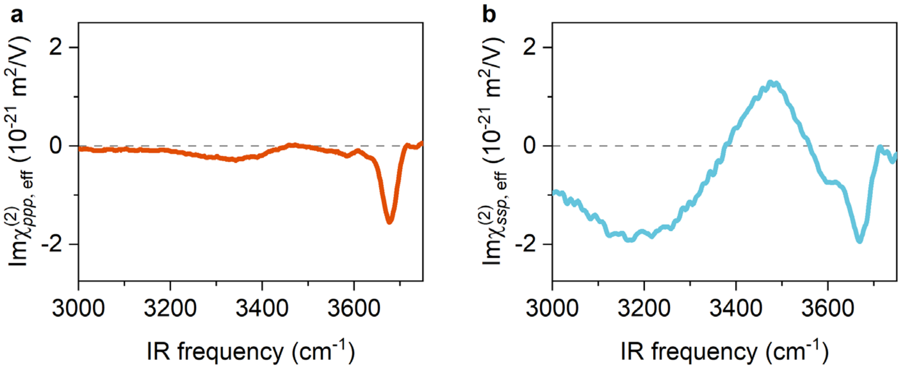

Fig. S24. *ssp* and *ppp* polarization analysis of the nanoconfined 3L water. a, b. The Im$\chi^{(2)}_{ppp,\,\mathrm{eff}}$, Im$\chi^{(2)}_{ssp,\,\mathrm{eff}}$ spectra. The grey dashed lines in (**a**) and (**b**) serve as zero lines.

S7. Amplitude Calibration of the SFG Spectra at ssp Polarization

The nanoconfined water sample HD-SFG spectra at *ssp* polarization were normalized with that from the gold at *ssp* polarization according to Eq. S4. To obtain the absolute value of the effective surface non-linear susceptibilities ($\chi^{(2)}_{ssp,\,\text{eff, sample}}$), one needs to calibrate the amplitude of the normalized spectra ($\chi^{(2)}_{ssp,\,\text{measured}}$) via:

$$\chi^{(2)}_{ssp,\,\text{eff, sample}} = \chi^{(2)}_{ssp,\,\text{measured}} \frac{r^{*}_{\text{CaF}_2\text{/gold}} \chi^{(2)}_{ssp,\,\text{eff, CaF}_2\text{/gold}}}{r^{*}_{\text{sample}}}, \tag{S14}$$

where $\chi^{(2)}_{ssp,\,\text{eff, CaF}_2\text{/gold}}$ is the effective SFG signal at the CaF_2/gold interface, $r^{*}_{\text{CaF}_2\text{/gold}}$ and r^{*}_{sample} are the reflectivity coefficients for the *s*-polarized LO beams at the CaF_2/gold interface and CaF_2/water/graphene interface, respectively. The reflectivity coefficients can be calculated via Eq. S15. In this study, the CaF_2/water/graphene interface was regarded as a CaF_2/air interface for the calculation (see Section S8 for details).

$$r = \frac{n_i \cos\theta_i - n_j \cos\theta_j}{n_i \cos\theta_i + n_j \cos\theta_j}, \tag{S15}$$

where θ_i and θ_j are the refracted angle of corresponding light (SF, vis, IR) in bulk medium *i* and *j*. n_i and n_j are the refractive index of corresponding light in bulk medium *i* and *j*.

To get $\chi^{(2)}_{ssp,\,\text{eff, sample}}$, one needs to know the result of $r^{*}_{\text{CaF}_2\text{/gold}} \chi^{(2)}_{ssp,\,\text{eff, CaF}_2\text{/gold}}$ which is an unknown value. Here, we obtained the $r^{*}_{\text{CaF}_2\text{/gold}} \chi^{(2)}_{ssp,\,\text{eff, CaF}_2\text{/gold}}$ in the following three steps:

1. To obtain the absolute value of the effective surface non-linear susceptibilities at the air/D_2O interface ($\chi^{(2)}_{ssp,\,\text{eff, air/D}_2\text{O}}$), we calibrated the amplitude of the measured normalized spectra ($\chi^{(2)}_{ssp,\,\text{air/D}_2\text{O}}$) via:

$$\chi^{(2)}_{ssp,\,\text{eff, air/D}_2\text{O}} = i\chi^{(2)}_{ssp,\,\text{air/D}_2\text{O}} \frac{r^{*}_{\text{air/zqz}} \left|\chi^{(2)}_{ssp,\,\text{eff, air/zqz}}\right|}{r^{*}_{\text{air/D}_2\text{O}}}, \tag{S16}$$

where $r^{*}_{\text{air/D}_2\text{O}}$ and $r^{*}_{\text{air/zqz}}$ are the reflectivity coefficients of the *s*-polarized LO beams at the air/D_2O interface and the air/zqz interface, respectively. $\chi^{(2)}_{ssp,\,\text{air/D}_2\text{O}}$ is the measured spectrum at the air/D_2O interface normalized by the signal at the air/zqz interface. $\chi^{(2)}_{ssp,\,\text{eff, air/zqz}}$ is the effective

SFG signal at the air/zqz interface which is a known value and can be calculated from the parameters listed in Tables S1 and Eq. S17[59,62]. For H_2O, frequency-dependent refractive index was employed[62,63].

$$\chi^{(2)}_{ssp,\text{ eff, air/zqz}} = 2L_{yy}(\omega_{\text{SF}})L_{yy}(\omega_{\text{vis}})L_{xx}(\omega_{\text{IR}}) \cos\theta_i(\omega_{\text{IR}})\chi^{(2)}_q\, l_c, \tag{S17}$$

where $\chi^{(2)}_q \approx 8\times10^{-13}\text{mV}^{-1}$ is the second-order susceptibility of the zqz. ω_{SF}, ω_{vis}, and ω_{IR} are the frequency of the corresponding beam, respectively. θ_i is the incident angle of the IR beam in bulk medium i as shown in Fig. S25. L_{ii} $(i = x, y, z)$ is the ii component of the Fresnel coefficients, and is given by:

$$L_{xx} = \frac{2n_i\cos\theta_j}{n_i\cos\theta_j + n_j\cos\theta_i}, \tag{S18}$$

$$L_{yy} = \frac{2n_i\cos\theta_i}{n_i\cos\theta_i + n_j\cos\theta_j}, \tag{S19}$$

$$L_{zz} = \frac{2n_j\cos\theta_i}{n_j\cos\theta_i + n_i\cos\theta_j}\frac{n_i^2}{n'^2}, \tag{S20}$$

where n' is the refractive index of the interfacial layer. Detailed discussion on n' can be found in Section S8. The beam configuration of the SFG measurement is displayed in Fig. S25.

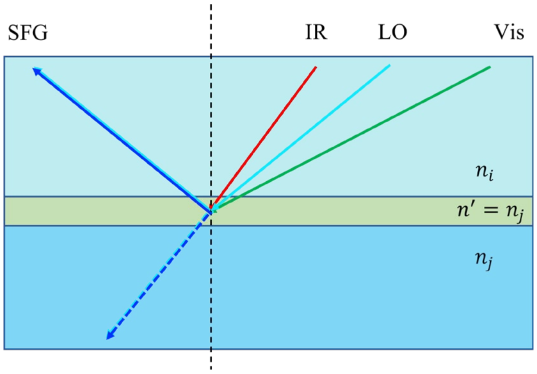

Fig. S25. The beam configuration of the non-colinear HD-SFG measurement.

The effective coherence length for the reflected SFG, l_c is calculated by:

$$l_c = \frac{1}{k_{2z}(\omega_{\text{SF}}) + k_{2z}(\omega_{\text{vis}}) + k_{2z}(\omega_{\text{IR}})} \approx 43\text{ nm}, \tag{S21}$$

where k_{2z} is the wavevector of corresponding lights in medium j along the z-axis. The incidence angles of IR and visible beams in air are 50° and 64°, respectively. By using Eqs. S17-S21 and the parameters listed in Tables S1, we obtain $\chi^{(2)}_{ssp,\text{ eff, air/zqz}}$. From Eq. S16, we obtain $\chi^{(2)}_{ssp,\text{ eff, air/D}_2\text{O}}$.

2. Assuming that $\chi^{(2)}_{yyz,\text{ CaF}_2\text{/D}_2\text{O}} = -\chi^{(2)}_{yyz,\text{ air/D}_2\text{O}}$, one can get:

$$\frac{\chi^{(2)}_{ssp,\text{ eff, CaF}_2\text{/D}_2\text{O}}}{F^{\text{CaF}_2\text{/D}_2\text{O}}} = -\frac{\chi^{(2)}_{ssp,\text{ eff, air/D}_2\text{O}}}{F^{\text{air/D}_2\text{O}}}, \tag{S22}$$

where $\chi^{(2)}_{ssp,\text{ eff, CaF}_2\text{/D}_2\text{O}}$ is the effective nonresonant SFG signal from the CaF_2/D_2O interface. F is the Fresnel factor and is given by Eq. S23. Using Eqs. S22-S23, we obtain $\chi^{(2)}_{ssp,\text{ eff, CaF}_2\text{/D}_2\text{O}}$.

$$F = L_{yy}(\omega_{\text{SF}})L_{yy}(\omega_{\text{vis}})L_{zz}(\omega_{\text{IR}})\sin\theta_i(\omega_{\text{IR}})\,. \tag{S23}$$

The assumption that $\chi^{(2)}_{yyz,\text{ CaF}_2\text{/D}_2\text{O}} = -\chi^{(2)}_{yyz,\text{ air/D}_2\text{O}}$ is based on the fact that the non-resonant response primarily originates from the quadrupole contribution[64,65], which depends on the electric field gradient across the interface rather than directly on the field intensity at the interface. The sign of the electric field gradient reverses between the CaF_2/D_2O interface and the air/D_2O interface, leading to a difference in the sign of their respective non-resonant signals. However, the amplitude of the non-resonant signal is largely insensitive to the interface type. To support this, we compared the $\chi^{(2)}_{yyz}$ signals measured at the air/D_2O and lipid(DPPG)/D_2O interfaces. As shown in Fig. S26a, the signal for air/D_2O is purely real, spectrally flat in the O-H stretching frequency region, consistent with previous study[14]. Importantly, the signal for lipid/D_2O interface exhibit almost the same lineshape and amplitude, indicating that the non-resonant signals are identical at both interfaces. To further investigate the influence of refractive index and chemical nature of the solid, we examined the signals at SiO_2/D_2O, CaF_2/D_2O, and Al_2O_3/D_2O interfaces, where the solids have refractive indices of 1.41, 1.42, and 1.71 for IR light (~3 μm), respectively, and surface charges that are negative, positive, and nearly neutral at pH ~6. These signals are also spectrally flat in the O-H stretching frequency region, consistent with previous studies[16,19,66,67], and within experimental uncertainty, exhibit the same (real) amplitude as those at the air/D_2O and DPPG/D_2O interfaces (Fig. S26b). These findings indicate that the non-resonant signal is also insensitive to both the refractive index and the chemical nature of the solid. Notably, previous experimental studies on various air/liquid interfaces have demonstrated that the non-resonant signal remains

largely unaffected by the type of liquid[64], which aligns with our observations. We therefore conclude that this approximation is valid and does not compromise phase accuracy.

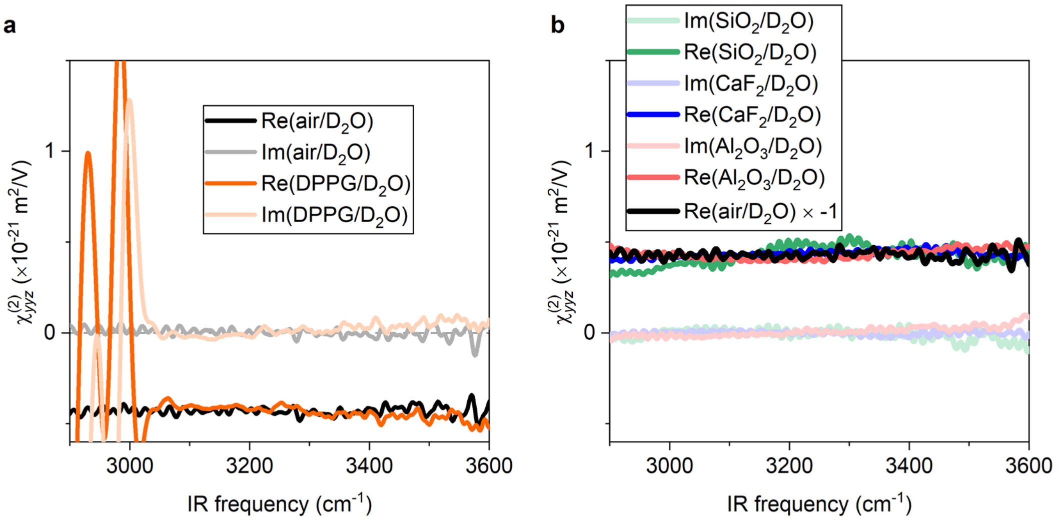

Fig. S26. Non-resonant signal. a. Experimental Im$\chi_{yyz}^{(2)}$ and Re$\chi_{yyz}^{(2)}$ spectra in the O-H stretching frequency region at the air/D_2O and DPPG/D_2O interfaces. **b**. Experimental Im$\chi_{yyz}^{(2)}$ and Re$\chi_{yyz}^{(2)}$ spectra at the SiO_2/D_2O, CaF_2/D_2O, and Al_2O_3/D_2O interfaces. For comparison, the $-$Re$\chi_{yyz}^{(2)}$ spectrum of the air/D_2O interface is also shown in (**b**). The dashed grey lines in (**a**) and (**b**) serve as zero lines.

3. To obtain the absolute value of the effective surface non-linear susceptibilities at the CaF_2/gold interface ($r^*_{\mathrm{CaF_2/gold}}\chi^{(2)}_{ssp,\,\mathrm{eff,\,CaF_2/gold}}$), we calibrate the amplitude of the measured normalized spectra ($\chi^{(2)}_{ssp,\,\mathrm{CaF_2/D_2O}}$) via:

$$r^*_{\mathrm{CaF_2/gold}}\chi^{(2)}_{ssp,\,\mathrm{eff,\,CaF_2/gold}} = \frac{r^*_{\mathrm{CaF_2/D_2O}}\chi^{(2)}_{ssp,\,\mathrm{eff,\,CaF_2/D_2O}}}{\chi^{(2)}_{ssp,\,\mathrm{CaF_2/D_2O}}}, \tag{S24}$$

where $r^*_{\mathrm{CaF_2/D_2O}}$ is the reflectivity of LO at the CaF_2/D_2O interface. $\chi^{(2)}_{ssp,\,\mathrm{CaF_2/D_2O}}$ is the measured spectrum at the CaF_2/D_2O interface normalized by the signal at the CaF_2/gold interface. From these three steps, we finally obtain:

$$r^*_{\mathrm{CaF_2/gold}}\chi^{(2)}_{ssp,\,\mathrm{eff,\,CaF_2/gold}} \approx 3.2 \times 10^{-20}\ \mathrm{m^2V^{-1}}. \tag{S25}$$

Table S1. Refractive Indexes Used to Calculate the Fresnel Factors.

Refractive index *n*	SF (~635 nm)	Vis (800 nm)	IR (3300 nm)
CaF_2	1.43	1.43	1.42
SiO_2	1.46	1.45	1.41
D_2O	1.33	1.33	1.25
zqz	1.54	1.54	1.52

S8. Extraction of $\chi_{yyz}^{(2)}$ from $\chi_{ssp,\,\mathrm{eff}}^{(2)}$

The effective SFG signal ($\chi_{ssp,\,\mathrm{eff}}^{(2)}$) at the *ssp* polarization is given by:

$$\chi_{ssp,\,\mathrm{eff}}^{(2)} = F\chi_{yyz}^{(2)}. \tag{S26}$$

Note that the interfacial dielectric constant is critical to do the Fresnel factor corrections and to extract the amplitude of the *yyz* components of the measured $\chi^{(2)}$ spectra ($\chi_{yyz}^{(2)}$). Two homogeneous interfacial dielectric constant models, the Lorentz model ($n' = n_j$) and the Slab model ($n' = \sqrt{\frac{n_i^2(n_i^2+5)}{4n_i^2+2}}$), are usually employed[59,60]. Such corrections and underlying assumptions of the interfacial dielectric constant[62,68] critically affect the SFG amplitude. For the *ssp* polarization combination, our recent studies show that the Lorentz model allows for more accurate Fresnel factor corrections at aqueous interfaces[62], while the slab model underestimates the H-bonded O-H stretch signal. Therefore, in this work, the Lorentz model is employed to do the Fresnel factor corrections at all different sample geometries.

For the air/suspended graphene/water interface, we consider it as the air/water interface to do the Fresnel factor corrections because the monolayer graphene with one-atom thickness hardly affects the corrections[69]. The interface layer for the nanoconfined water sample is intricate, comprising two interfaces: CaF_2/confined water and confined water/graphene interfaces, which raises the question of whether one homogeneous interfacial dielectric constant can accurately capture the interface layer. Indeed, the effective dielectric constant of the nanoconfined water is unclear. To address this question, we measured the conventional SFG signals ($\left|\chi_{ssp,\,\mathrm{eff,\,CaF_2/water/graphene}}^{(2)}\right|^2$) at the CaF_2/confined water/graphene interface through normalization with CaF_2/gold signal, and compared it with the signal ($\left|\chi_{ssp,\,\mathrm{eff,\,graphene/water/CaF_2}}^{(2)}\right|^2$) obtained from the same sample but with graphene positioned on top and normalization with air/gold signal. In both sample geometries, the spectral lineshape remains consistent. After eliminating the Fresnel factor using Eqs. S23 and S26 by considering a CaF_2/air interface and air/CaF_2 interface, respectively, the spectral amplitudes are also consistent (Fig. S27). These results indicate that one homogeneous interfacial dielectric constant is sufficient to capture the sub-nanometer thickness interface layer, and the Lorentz model is accurate for the Fresnel factor corrections for the nanoconfined water samples.

We calculated the Fresnel factor for the CaF_2/water/graphene interface by treating it as a CaF_2/air interface. This approach is justified because the thickness of the interfacial layer (the nanoconfined water layer combined with the graphene monolayer) is approximately 1 nm, which is significantly smaller than the wavelengths of the SFG, visible, and IR light. Consequently, this thin layer does not introduce any noticeable changes in the effective reflection and transmission of these lights at the interface, and therefore does not affect the Fresnel factor (both amplitude and phase), as discussed in Refs.[69,70]. We note that the propagation phase change of the three lights (SFG, visible, and IR) arising from the presence of a ~1 nm interfacial layer is also negligibly small, less than 1° considering a three-layer dielectric model[69], with the Lorentz model to describe the interfacial layer. Therefore, the approximation does not cause a significant phase error.

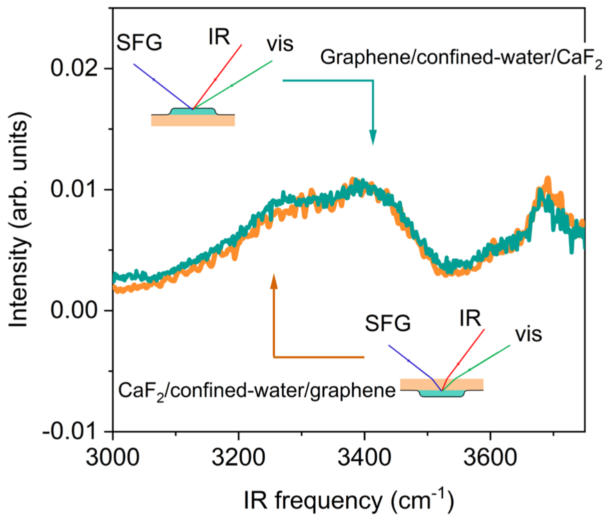

Fig. S27. The feasibility of the method for extracting $\chi^{(2)}_{yyz}$ from $\chi^{(2)}_{ssp}$. Conventional SFG signals at the CaF_2/confined-water/graphene interface and the graphene/confined-water/CaF_2 interface. The insets show the corresponding sample geometries and (SFG, vis, IR) beam geometries for the conventional SFG measurements.

S9. Real part of $\chi_{yyz}^{(2)}$

Fig. S28 shows the real part of the $\chi_{yyz}^{(2)}$ spectra ($\mathrm{Re}\chi_{yyz}^{(2)}$) of the nanoconfined three-layer water and its comparison with the sum of the water/graphene and CaF_2/water signals. Still, the Eq. (1) holds.

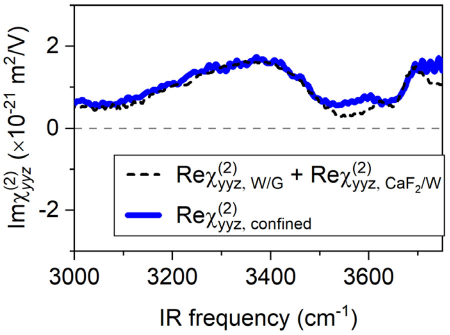

Fig. S28. Experimental $\mathrm{Re}\chi_{yyz}^{(2)}$ of nanoconfined three-layer water (blue solid line). The sum of the water/graphene and CaF_2/water signals obtained from HD-SFG experiments is also shown for comparison (black dashed line). A constant non-resonance contribution is subtracted in the summed spectrum because the non-resonance contribution is counted twice in the summed spectrum. Dashed grey line indicates a zero line.

S10. Structural Dynamic Information of Water Computed from AIMD Data

To examine whether the structure of the interfacial water is similar between the bulk water/graphene interface or the CaF_2/bulk water interface and the nanoconfined systems, we characterized the structure of the interfacial water in the following two ways; the joint probability of the two O-H group angles of water molecules and the depth profiles of the dipole orientation of water molecules. First, we calculated the joint probability distributions $P(\cos\theta_{OH_1}; \cos\theta_{OH_2})$ for the orientations of a water molecule's two O-H bond vectors[71], where θ_{OH_1} is the angle formed by one O-H bond of a water molecule and the surface normal (*z*-axis) and θ_{OH_2} is the angle formed by the other O-H bond of the water molecule and *z*-axis. The data for the topmost water layer near the graphene sheet and near the CaF_2 surface are shown in Fig. S29 and S30, respectively. Fig. S29 shows that the angle distributions of the water are similar between the bulk water/graphene system and the nanoconfined water systems. A similar observation is obtained for the data near the CaF_2 surface (Fig. S30). This further confirms that the structure of the ~1 nm interfacial water systems and nanoconfined water systems closely resemble, in good agreement with the conclusion obtained from the SFG spectra. Furthermore, we computed the dipole orientation profiles. The data shown in Fig. S31 further confirms the notation that the interfacial water system and nanoconfined water systems are similar. We also calculated the anisotropy decay time[72] of the free OH group in water molecules near each surface for all bulk interfaces and nanoconfined water systems. The data shown in Fig. S32 indicates that the interfacial and nanoconfined water systems exhibit similar dynamic properties.

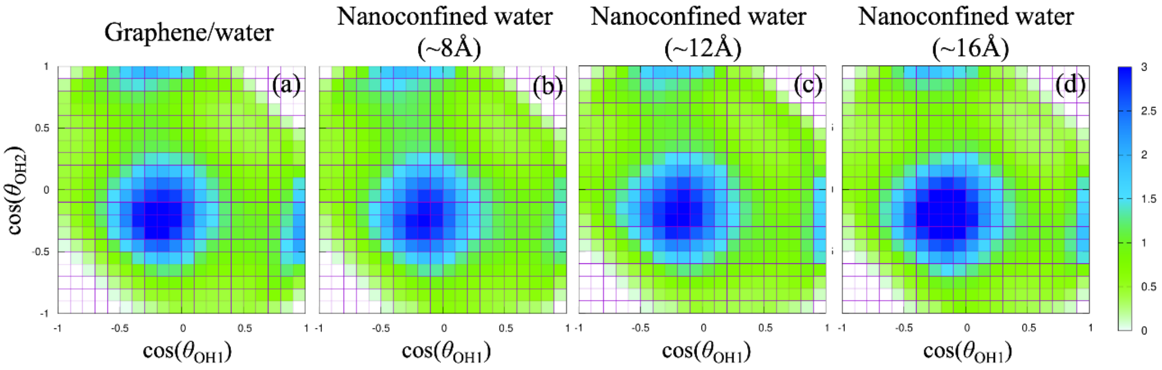

Fig. S29. Joint probability distributions $P(\cos\theta_{OH_1}; \cos\theta_{OH_2})$ for the orientations of the topmost layer of water molecule's two OH bond vectors near the graphene sheet computed from the AIMD data. A. The water/graphene interface system. **b-d**, the nanoconfined systems

with water thickness of ~ 8 Å (**b**), ~ 12 Å (**c**), and ~ 16 Å (**d**). The topmost layer was defined as 0 Å < z < 4.7 Å, according to the density minima of the number density of water shown in Fig. S9. The origin point of the *z*-axis was the position of the graphene sheet. All the plots are normalized by the probability distribution for the corresponding isotopic bulk environment.

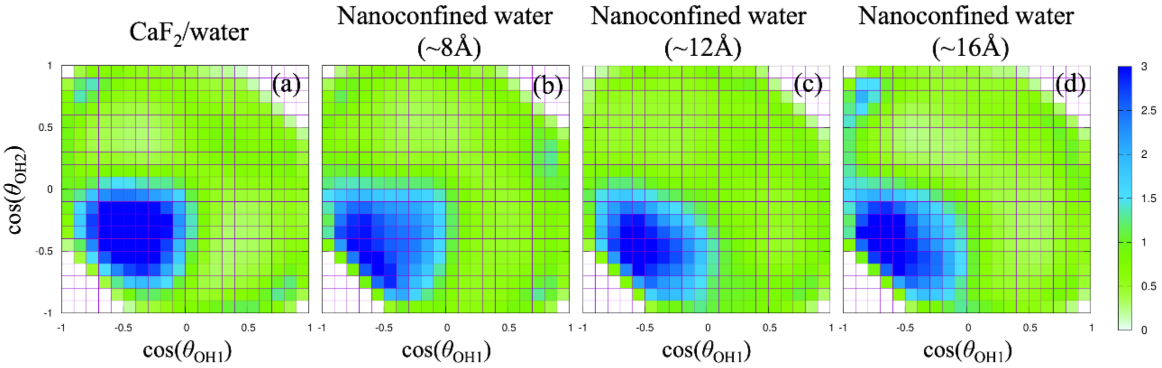

Fig. S30. Joint probability distributions $P(\cos\theta_{OH_1};\cos\theta_{OH_2})$ for the orientations of the topmost layer of water molecule's two OH bond vectors near the CaF_2 surface computed from the AIMD data. a. The CaF_2/water interface system. **b-d**, the nanoconfined systems with water thickness of ~ 8 Å (**b**), ~ 12 Å (**c**), and ~ 16 Å (**d**). The topmost layer for the CaF_2/water interface system was defined as -3.5 Å < *z*' < 0 Å while the topmost layers were defined as 7.3 Å < *z* < 10.8 Å, 11.3 Å < *z* < 14.8 Å and 15.3 Å < *z* < 18.8 Å for the nanoconfined water systems with their thickness of ~8 Å, ~12 Å, and ~16 Å, respectively. *z* (*z*')-axis is the surface normal whose origin point is at the graphene position (first layer F atom position), according to the density minima of the number density of water shown in Fig. S9. All the plots are normalized by the probability distribution for the corresponding isotopic bulk environment.

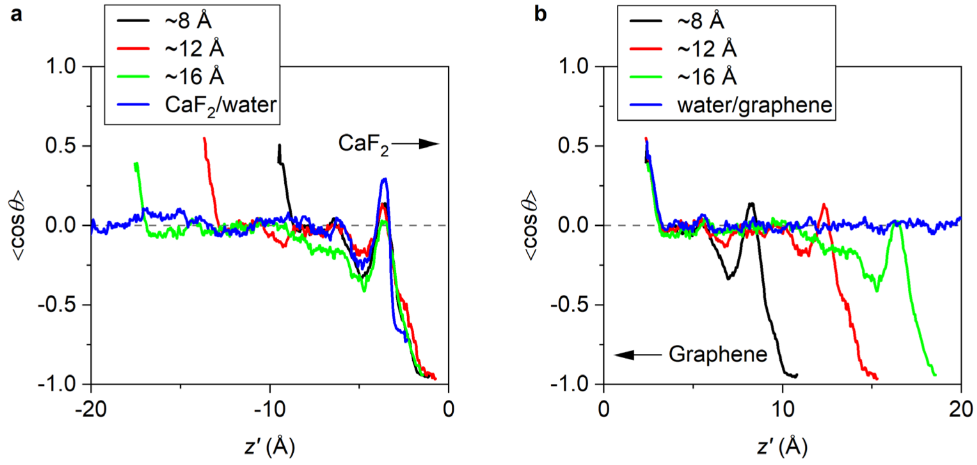

Fig. S31. Depth profiles of the dipole orientation of water computed by using AIMD data. a, the CaF_2 surface side. **b**, the graphene sheet side. The angle θ is defined as the angle between the bisector of a water molecule and the surface normal (*z*- or *z*'-axis).

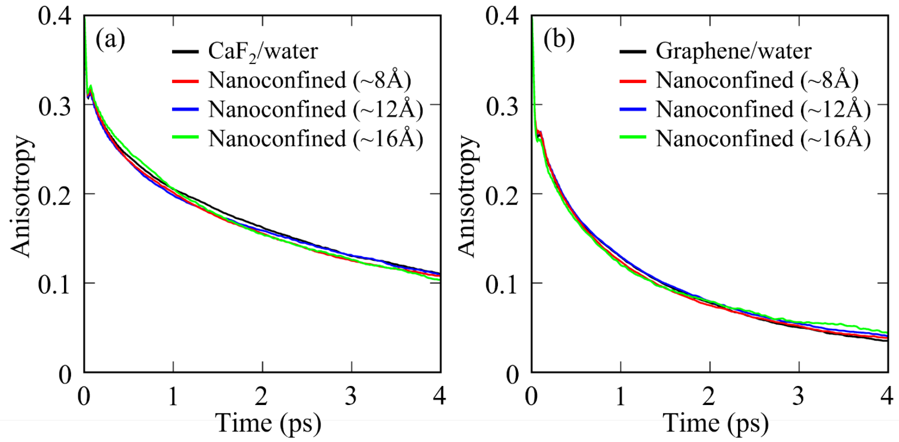

Fig. S32. The anisotropy decay time of the free OH group of the water molecules in the top layer near the a, the CaF_2 surface side. b, the graphene sheet side. The topmost layer near CaF_2/water interface system was defined as -3.5 Å < *z*' < 0 Å while the topmost layers near the CaF_2 surface side were defined as 7.3 Å < *z* < 10.8 Å, 11.3 Å < *z* < 14.8 Å and 15.3 Å < *z* < 18.8 Å for the nanoconfined water systems with their thickness of ~8 Å, ~12 Å, and ~16 Å, respectively. The topmost layer near the graphene sheet side was defined as 0 Å < z < 4.7 Å, according to the density minima of the number density of water shown in Fig. S9. *z* (*z*')-axis is the surface normal whose origin point is at the graphene position (first layer F atom position), according to the density minima of the number density of water shown in Fig. S9.

S11. Solubility of LiCl Ions under Nanoconfinement

Upon decreasing the thickness of the nanoconfined water, the ion concentration in the confinement may increase by 1-2 fold but it is still below the solubility limit of LiCl (~18 M). To confirm the notion and show that the in-plane distribution of salt concentration between the graphene and the CaF_2 substrate remains uniform upon decreasing the RH, we conducted AFM measurements at different RHs. The AFM height images of the nanoconfined water between the graphene sheet and CaF_2 substrate are presented in Figs. S33a and b. As the RH decreases from ~25% to ~5%, the surface roughness of the sample shows only weak change, measuring 1.2 Å and 1.3 Å, and no significant precipitation of LiCl is observed. This confirms that the confined LiCl concentration remains below the solubility limit of LiCl, consistent with our estimation that the LiCl concentration of the ~8 Å confined water is around 2-3 M (see Section S1), which may increase to ~6-7 M as the RH decreases to ~5%. Furthermore, we increase the RH back to ~25%, confirming that the $\mathrm{Im}\chi^{(2)}_{yyz}$ spectrum is reversible (Fig. S33c). This observation provides additional evidence supporting our statement.

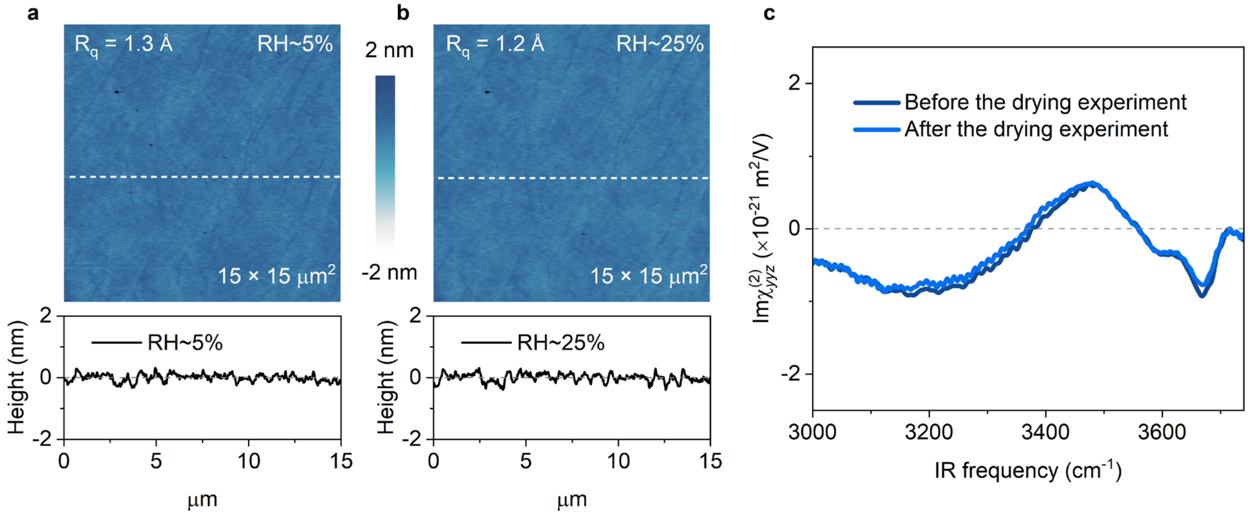

Fig. S33. In-plane distribution of salt concentration. a, **b**. AFM height image of the nanoconfined water at (**a**) RH~5% and (**b**) RH~25%. The bottom panel in each AFM height image shows the typical height profiles along the white dashed lines in the corresponding AFM height image. **c**. Reversible experimental $\mathrm{Im}\chi^{(2)}_{yyz}$ spectra of the nanoconfined water upon RH change. The dashed line serves as a zero line. The two spectra were measured at RH~25%.

S12. Dependence of Water Thickness in Nanoconfined System on SFG Spectra

We further examined the dependence of the water thickness confined by the graphene sheet and the CaF_2 substrate on the SFG spectra. To this end, we computed the SFG signals with different fractions of water in the nanoconfined systems. The data are shown in Fig. S34. One can see that the variation of the SFG signal is rather insensitive to the fraction of the water molecules, consistent with the experimental data, and is also consistent with the previous theoretical prediction[73]. This insensitivity of the SFG signal to the water thickness manifests that the thickness of the water layer more than ~8 Å does not affect the SFG signal for the high ion concentration even though the CaF_2 surface is charged. In fact, the Debye length for the 1 M ion concentration is ~3 Å, consistent with our observation.

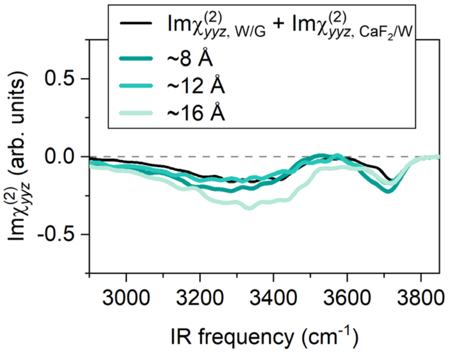

Fig. S34. Simulated $\mathrm{Im}\chi^{(2)}_{yyz}$ spectra of nanoconfined water of different thicknesses computed from AIMD data. The dashed line indicates the zero line.

S13. SFG Spectra Computed from AIMD Data

Here, we showed the series of SFG spectra computed from the AIMD trajectories. The snapshot is shown in Fig. S35a, while the computed spectra are shown in Figs. S35b-d for the confined water system, water/graphene interface, and CaF_2/water interface, respectively. Since the AIMD trajectories are much shorter than the MLFF-MD trajectories, the s/n ratio is worse in the spectra computed from the AIMD trajectory than those computed from the MLFF-MD trajectories for the CaF_2/water interface and the nanoconfined water system. Nevertheless, within the noise of the spectra, Eq. (1) in the main text is valid, meaning that the nanoconfined SFG spectrum is composed of the SFG spectra at the CaF_2/water interface and the water/graphene interface.

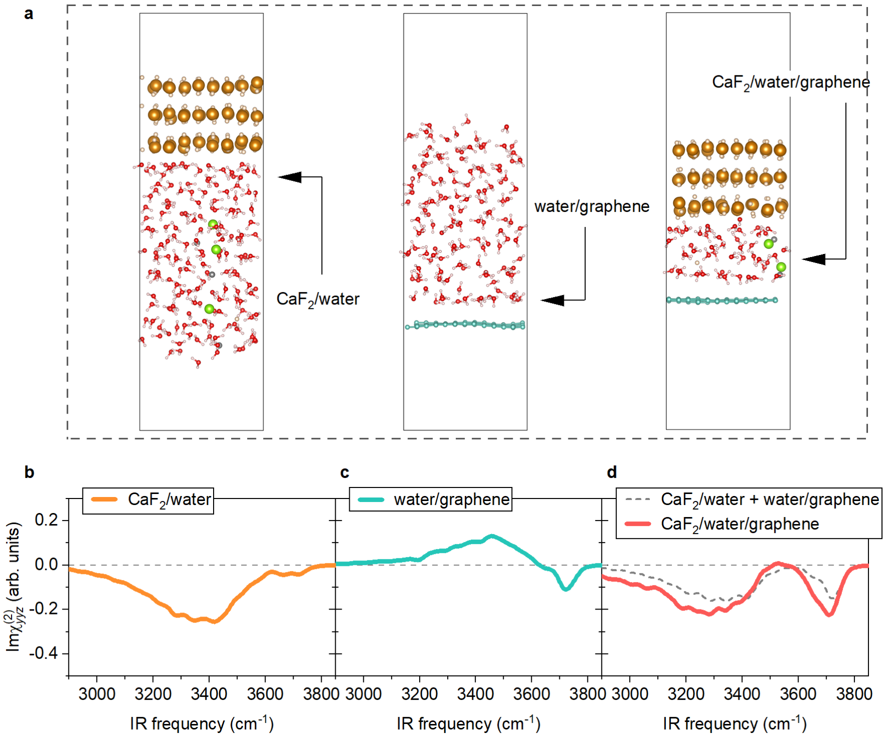

Fig. S35. SFG spectra calculated from AIMD. a. Snapshots of the CaF_2/water interface, water/graphene interface, and nanoconfined water obtained from the AIMD simulation. The yellow, light yellow, red, light pink, green, grey, and cyan spheres indicate the Ca, F, O, H, Cl, Li, and C atoms, respectively. **b**, **c**, **d**. AIMD based $\mathrm{Im}\chi^{(2)}_{yyz}$ spectra of (**b**) the CaF_2/water interface; (**c**) the water/graphene interface; and (**d**) nanoconfined water (red line). The sum of the CaF_2/water (**b**) and water/graphene (**c**) SFG signals is shown in (**d**) for comparison (black line). Dashed lines in (**b-d**) indicate zero lines.

S14. SFG Spectra Computed from AIMD and MLFF-MD Trajectories

To check the reproducibility of the MLFF-MD data, we performed the MLFF-MD at the CaF_2(111)/water interface for the cell with its size of $\vec{a}$, $\vec{b}$, and $\vec{c}$. The composition of the system is the same between the AIMD and MLFF-MD. The resulting depth profile of the dipole orientation of water and SFG spectra of water are shown in Fig. S36. This figure displays good agreement between the AIMD and MLFF-MD data, manifesting that the MLFF-MD can capture the trend of the AIMD data. With the current ion concentration in our study, as the ions may screen the long-range Coulomb interactions, the long-range effects may not be important. Therefore, our MLFF-MD spectra data without long-range Coulomb interactions agrees with AIMD data, this is in line with previous studies, where they did not find significant changes in spectral properties due to the long-range corrections[74–76].

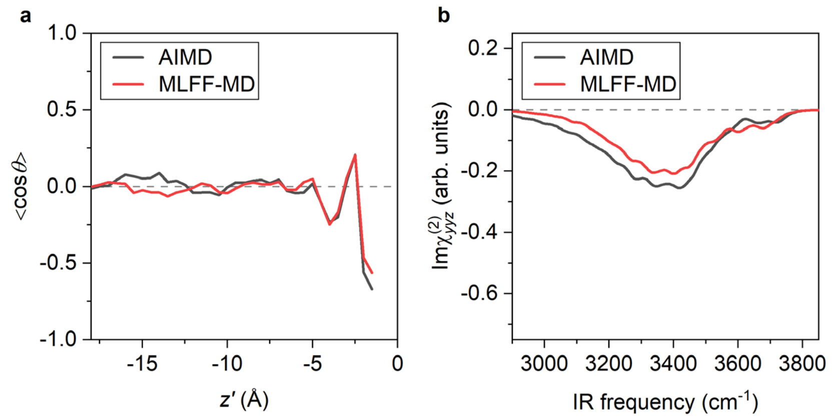

Fig. S36. Comparison of the AIMD data and MLFF-MD data at the CaF_2/water interface generated by the AIMD trajectories and MLFF-MD trajectories. a, **b**. Dipole orientation (**a**) of water along the surface normal and simulated $\mathrm{Im}\chi^{(2)}_{yyz}$ spectra (**b**) of water.

S15. The Ions Distributions of the Nanoconfined System from MLFF-MD Trajectories

The ion distribution can give insights into the confined water structure. Fig. S37 shows the ion distributions of Li^+ and Cl^- of the nanoconfined system (~8 Å) with high (6 M) and low (2 M) ion concentrations calculated by the MLFF molecular dynamics simulation. The ions accumulate near the charged CaF_2 surface with their population gradually diminishing towards zero near the charged-neutral graphene side. Despite the different ion concentrations, the SFG spectra remain unaffected, as shown in Fig. S15d.

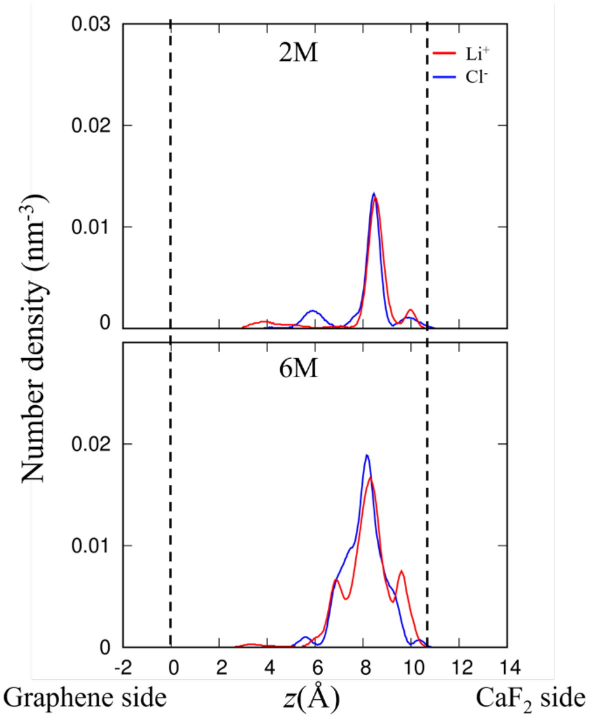

Fig. S37. Ion distribution along the surface normal for the nanoconfined system (~8 Å) with high (6 M) and low (2 M) ion concentrations calculated by the MLFF-MD simulation. The dash lines marked the position of the first layer of the atoms at the graphene or CaF_2 sides.

Supplementary References

1. Xu, K., Cao, P. & Heath, J. R. Graphene visualizes the first water adlayers on mica at ambient conditions. *Science* **329**, 1188–1191 (2010).
2. Li, Q., Song, J., Besenbacher, F. & Dong, M. Two-dimensional material confined water. *Acc. Chem. Res.* **48**, 119–127 (2015).
3. Yang, Q. *et al.* Capillary condensation under atomic-scale confinement. *Nature* **588**, 250–253 (2020).
4. Xu, Y., Ma, Y.-B., Gu, F., Yang, S.-S. & Tian, C.-S. Structure evolution at the gate-tunable suspended graphene–water interface. *Nature* **621**, 506–510 (2023).
5. Wang, Y. *et al.* Heterodyne-detected sum-frequency generation vibrational spectroscopy reveals aqueous molecular structure at the suspended graphene/water interface. *Angew. Chem. Int. Ed.* **63**, e202319503 (2024).
6. Yang, S. *et al.* Nature of the electrical double layer on suspended graphene electrodes. *J. Am. Chem. Soc.* **144**, 13327–13333 (2022).
7. Wang, Y. *et al.* Chemistry governs water organization at a graphene electrode. *Nature* **615**, E1–E2 (2023).
8. Wang, Y. *et al.* Direct probe of electrochemical pseudocapacitive ph jump at a graphene electrode**. *Angew. Chem. Int. Ed.* **62**, e202216604 (2023).
9. Takeshita, N., Okuno, M. & Ishibashi, T. Molecular conformation of DPPC phospholipid Langmuir and Langmuir–Blodgett monolayers studied by heterodyne-detected vibrational sum frequency generation spectroscopy. *Phys. Chem. Chem. Phys.* **19**, 2060–2066 (2017).
10. Nihonyanagi, S. (二本柳聡史), Yamaguchi, S. (山口祥一) & Tahara, T. (田原太平). Direct evidence for orientational flip-flop of water molecules at charged interfaces: A heterodyne-detected vibrational sum frequency generation study. *J. Chem. Phys.* **130**, 204704 (2009).
11. Vanselous, H. & Petersen, P. B. Extending the capabilities of heterodyne-detected sum-frequency generation spectroscopy: probing any interface in any polarization combination. *J. Phys. Chem. C* **120**, 8175–8184 (2016).
12. Takeshita, N., Okuno, M. & Ishibashi, T. Development of heterodyne-detected total internal reflection vibrational sum frequency generation spectroscopy and its application to caf2/liquid interfaces. *J. Phys. Chem. C* **121**, 25206–25214 (2017).
13. Urashima, S., Myalitsin, A., Nihonyanagi, S. & Tahara, T. The topmost water structure at a charged silica/aqueous interface revealed by heterodyne-detected vibrational sum frequency generation spectroscopy. *J. Phys. Chem. Lett.* **9**, 4109–4114 (2018).
14. Adhikari, A. Accurate determination of complex $\chi^{(2)}$ spectrum of the air/water interface. *J. Chem. Phys.* **143**, 124707 (2015).
15. Hu, X.-H., Wei, F., Wang, H. & Wang, H.-F. α-Quartz crystal as absolute intensity and phase standard in sum-frequency generation vibrational spectroscopy. *J. Phys. Chem. C* **123**, 15071–15086 (2019).
16. Dalstein, L., Potapova, E. & Tyrode, E. The elusive silica/water interface: isolated silanols under water as revealed by vibrational sum frequency spectroscopy. *Phys. Chem. Chem. Phys.* **19**, 10343–10349 (2017).
17. Wei, F., Urashima, S., Nihonyanagi, S. & Tahara, T. Elucidation of the ph-dependent electric double layer structure at the silica/water interface using heterodyne-detected vibrational sum frequency generation spectroscopy. *J. Am. Chem. Soc.* **145**, 8833–8846 (2023).
18. Hunger, J. *et al.* Nature of cations critically affects water at the negatively charged silica interface. *J. Am. Chem. Soc.* **144**, 19726–19738 (2022).
19. Piontek, S. M. *et al.* Probing the gold/water interface with surface-specific spectroscopy. *ACS Phys. Chem Au* **3**, 119–129 (2023).
20. Kühne, T. D. *et al.* CP2K: An electronic structure and molecular dynamics software package - Quickstep: Efficient and accurate electronic structure calculations. *J. Chem. Phys.* **152**, 194103 (2020).

21. Hutter, J., Iannuzzi, M., Schiffmann, F. & VandeVondele, J. cp2k: atomistic simulations of condensed matter systems. *WIREs Comput. Mol. Sci.* **4**, 15–25 (2014).

22. Puchin, V. E., Puchina, A. V., Huisinga, M. & Reichling, M. Theoretical modelling of steps on the CaF2(111) surface. *J. Phys.: Condens. Matter* **13**, 2081 (2001).

23. Perdew, J. P., Burke, K. & Ernzerhof, M. Generalized gradient approximation made simple. *Phys. Rev. Lett.* **77**, 3865–3868 (1996).

24. Zhang, Y. & Yang, W. Comment on "Generalized gradient approximation made simple". *Phys. Rev. Lett.* **80**, 890 (1998).

25. Grimme, S., Antony, J., Ehrlich, S. & Krieg, H. A consistent and accurate ab initio parametrization of density functional dispersion correction (DFT-D) for the 94 elements H-Pu. *J. Chem. Phys.* **132**, 154104 (2010).

26. Ohto, T. *et al.* Accessing the accuracy of density functional theory through structure and dynamics of the water–air interface. *J. Phys. Chem. Lett.* **10**, 4914–4919 (2019).

27. Goedecker, S., Teter, M. & Hutter, J. Separable dual-space Gaussian pseudopotentials. *Phys. Rev. B* **54**, 1703 (1996).

28. Krack, M. Pseudopotentials for H to Kr optimized for gradient-corrected exchange-correlation functionals. *Theor. Chem. Acc.* **114**, 145–152 (2005).

29. Bussi, G., Donadio, D. & Parrinello, M. Canonical sampling through velocity rescaling. *J. Chem. Phys.* **126**, 014101 (2007).

30. Bengtsson, L. Dipole correction for surface supercell calculations. *Phys. Rev. B* **59**, 12301–12304 (1999).

31. Becraft, K. A. & Richmond, G. L. In situ vibrational spectroscopic studies of the caf2/h2o interface. *Langmuir* **17**, 7721–7724 (2001).

32. Lis, D., Backus, E. H. G., Hunger, J., Parekh, S. H. & Bonn, M. Liquid flow along a solid surface reversibly alters interfacial chemistry. *Science* **344**, 1138–1142 (2014).

33. Cheng, J. & Sprik, M. Acidity of the aqueous rutile tio2(110) surface from density functional theory based molecular dynamics. *J. Chem. Theory Comput.* **6**, 880–889 (2010).

34. Zhang, L., Han, J., Wang, H., Car, R. & E, W. Deep potential molecular dynamics: a scalable model with the accuracy of quantum mechanics. *Phys. Rev. Lett.* **120**, 143001 (2018).

35. Wang, H., Zhang, L., Han, J. & E, W. DeePMD-kit: A deep learning package for many-body potential energy representation and molecular dynamics. *Comput. Phys. Commun.* **228**, 178–184 (2018).

36. Zeng, J. *et al.* DeePMD-kit v2: A software package for deep potential models. *J. Chem. Phys.* **159**, 054801 (2023).

37. Zhang, L., Lin, D.-Y., Wang, H., Car, R. & E, W. Active learning of uniformly accurate interatomic potentials for materials simulation. *Phys. Rev. Mater.* **3**, 023804 (2019).

38. Thompson, A. P. *et al.* LAMMPS - a flexible simulation tool for particle-based materials modeling at the atomic, meso, and continuum scales. *Comput. Phys. Commun.* **271**, 108171 (2022).

39. Ohto, T., Usui, K., Hasegawa, T., Bonn, M. & Nagata, Y. Toward ab initio molecular dynamics modeling for sum-frequency generation spectra; an efficient algorithm based on surface-specific velocity-velocity correlation function. *J. Chem. Phys.* **143**, 124702 (2015).

40. Auer, B. M. & Skinner, J. L. IR and Raman spectra of liquid water: Theory and interpretation. *J. Chem. Phys.* **128**, 224511 (2008).

41. Corcelli, S. A. & Skinner, J. L. Infrared and raman line shapes of dilute hod in liquid h2o and d2o from 10 to 90 °c. *J. Phys. Chem. A* **109**, 6154–6165 (2005).

42. Berens, P. H. & Wilson, K. R. Molecular dynamics and spectra. I. Diatomic rotation and vibration. *J. Chem. Phys.* **74**, 4872–4882 (1981).

43. Reddy, S. K. *et al.* Bulk contributions modulate the sum-frequency generation spectra of water on model sea-spray aerosols. *Chem* **4**, 1629–1644 (2018).

44. Wen, Y.-C. *et al.* Unveiling microscopic structures of charged water interfaces by surface-specific vibrational spectroscopy. *Phys. Rev. Lett.* **116**, 016101 (2016).

45. Ohno, P. E., Wang, H. & Geiger, F. M. Second-order spectral lineshapes from charged interfaces. *Nat. Commun.* **8**, 1032 (2017).

46. Schaefer, J., Gonella, G., Bonn, M. & Backus, E. H. G. Surface-specific vibrational spectroscopy of the water/silica interface: screening and interference. *Phys. Chem. Chem. Phys.* **19**, 16875–16880 (2017).

47. Hopkins, A. J., Schrödle, S. & Richmond, G. L. Specific ion effects of salt solutions at the caf $_2$ /water interface. *Langmuir* **26**, 10784–10790 (2010).

48. Gopinadhan, K. *et al.* Complete steric exclusion of ions and proton transport through confined monolayer water. *Science* **363**, 145–148 (2019).

49. Lee, J. E., Ahn, G., Shim, J., Lee, Y. S. & Ryu, S. Optical separation of mechanical strain from charge doping in graphene. *Nat. Commun.* **3**, 1024 (2012).

50. Calado, V. E., Schneider, G. F., Theulings, A. M. M. G., Dekker, C. & Vandersypen, L. M. K. Formation and control of wrinkles in graphene by the wedging transfer method. *Appl. Phys. Lett.* **101**, 103116 (2012).

51. Wang, C., Liu, Y., Lan, L. & Tan, H. Graphene wrinkling: formation, evolution and collapse. *Nanoscale* **5**, 4454–4461 (2013).

52. Vasu, K. S. *et al.* Van der Waals pressure and its effect on trapped interlayer molecules. *Nat. Commun.***7**, 12168 (2016).

53. Zabel, J. *et al.* Raman spectroscopy of graphene and bilayer under biaxial strain: bubbles and balloons. *Nano Lett.* **12**, 617–621 (2012).

54. Fisher, L. R., Gamble, R. A. & Middlehurst, J. The Kelvin equation and the capillary condensation of water. *Nature* **290**, (1981).

55. Yang, G., Chai, D., Fan, Z. & Li, X. Capillary condensation of single- and multicomponent fluids in nanopores. *Ind. Eng. Chem. Res.* **58**, 19302–19315 (2019).

56. Deroche, I., Daou, T. J., Picard, C. & Coasne, B. Reminiscent capillarity in subnanopores. *Nat. Commun.* **10**, 4642 (2019).

57. Song, R., Zou, T., Chen, J., Hou, X. & Han, X. Study on the physical properties of licl solution. *IOP Conf. Ser.: Mater. Sci. Eng.* **562**, 012102 (2019).

58. Kim, D. *et al.* Wettability of graphene and interfacial water structure. *Chem* **7**, 1602–1614 (2021).

59. Yu, C.-C. *et al.* Polarization-dependent heterodyne-detected sum-frequency generation spectroscopy as a tool to explore surface molecular orientation and ångström-scale depth profiling. *J. Phys. Chem. B* **126**, 6113–6124 (2022).

60. Chiang, K.-Y. *et al.* The dielectric function profile across the water interface through surface-specific vibrational spectroscopy and simulations. *Proc. Natl. Acad. Sci. U.S.A.* **119**, e2204156119 (2022).

61. Wang *, H.-F., Gan † ‡, W., Lu † ‡ §, R., Rao † ‡ ¶, Y. & Wu †, B.-H. Quantitative spectral and orientational analysis in surface sum frequency generation vibrational spectroscopy (SFG-VS). *Int. Rev. Phys. Chem.* **24**, 191–256 (2005).

62. Yu, X., Chiang, K.-Y., Yu, C.-C., Bonn, M. & Nagata, Y. On the Fresnel factor correction of sum-frequency generation spectra of interfacial water. *J. Chem. Phys.* **158**, 044701 (2023).

63. Hale, G. M. & Querry, M. R. Optical constants of water in the 200-nm to 200-μm wavelength region. *Appl. Opt., AO* **12**, 555–563 (1973).

64. Yamaguchi, S. (山口祥一), Shiratori, K. (白鳥和矢), Morita, A. (森田明弘) & Tahara, T. (田原太平). Electric quadrupole contribution to the nonresonant background of sum frequency generation at air/liquid interfaces. *J. Chem. Phys.* **134**, 184705 (2011).

65. Shen, Y. R. Revisiting the basic theory of sum-frequency generation. *J. Chem. Phys.* **153**, 180901 (2020).

66. Zhang, L., Tian, C., Waychunas, G. A. & Shen, Y. R. Structures and charging of α-alumina (0001)/water interfaces studied by sum-frequency vibrational spectroscopy. *J. Am. Chem. Soc.* **130**, 7686–7694 (2008).

67. Myalitsin, A., Urashima, S., Nihonyanagi, S., Yamaguchi, S. & Tahara, T. Water structure at the buried silica/aqueous interface studied by heterodyne-detected vibrational sum-frequency generation. *J. Phys. Chem. C* **120**, 9357–9363 (2016).

68. Zhuang, X., Miranda, P. B., Kim, D. & Shen, Y. R. Mapping molecular orientation and conformation at interfaces by surface nonlinear optics. *Phys. Rev. B* **59**, 12632–12640 (1999).

69. Moloney, E. G., Azam, Md. S., Cai, C. & Hore, D. K. Vibrational sum frequency spectroscopy of thin film interfaces. *Biointerphases* **17**, 051202 (2022).

70. Lu, X. *et al.* A sum frequency generation vibrational study of the interference effect in poly(n-butyl methacrylate) thin films sandwiched between silica and water. *J. Phys. Chem. C* **115**, 13759–13767 (2011).

71. Odendahl, N. L. & Geissler, P. L. Local ice-like structure at the liquid water surface. *J. Am. Chem. Soc.* **144**, 11178–11188 (2022).

72. Ohto, T., Tada, H. & Nagata, Y. Structure and dynamics of water at water–graphene and water–hexagonal boron-nitride sheet interfaces revealed by ab initio sum-frequency generation spectroscopy. *Phys. Chem. Chem. Phys.* **20**, 12979–12985 (2018).

73. Das, B., Ruiz-Barragan, S. & Marx, D. Deciphering the properties of nanoconfined aqueous solutions by vibrational sum frequency generation spectroscopy. *J. Phys. Chem. Lett.* **14**, 1208–1213 (2023).

74. Yue, S. *et al.* When do short-range atomistic machine-learning models fall short? *J. Chem. Phys.* **154**, 034111 (2021).

75. Omranpour, A., De Hijes, P. M., Behler, J. & Dellago, C. Perspective: atomistic simulations of water and aqueous systems with machine learning potentials. Preprint at https://doi.org/10.48550/arXiv.2401.17875 (2024).

76. Litman, Y., Lan, J., Nagata, Y. & Wilkins, D. M. Fully first-principles surface spectroscopy with machine learning. *J. Phys. Chem. Lett.* **14**, 8175–8182 (2023).
